# Chlamydiae as symbionts of photosynthetic dinoflagellates

**DOI:** 10.1093/ismejo/wrae139

**Published:** 2024-07-24

**Authors:** Justin Maire, Astrid Collingro, Kshitij Tandon, Vanta J Jameson, Louise M Judd, Matthias Horn, Linda L Blackall, Madeleine J H van Oppen

**Affiliations:** School of Biosciences, The University of Melbourne, Parkville, VIC 3010, Australia; Centre for Microbiology and Environmental Systems Science, University of Vienna, Vienna 1030, Austria; School of Biosciences, The University of Melbourne, Parkville, VIC 3010, Australia; Department of Microbiology and Immunology, The University of Melbourne at The Peter Doherty Institute of Infection and Immunity, Parkville, VIC 3010, Australia; Melbourne Cytometry Platform, The University of Melbourne, Parkville, VIC 3010, Australia; Doherty Applied Microbial Genomics, Department of Microbiology and Immunology, The University of Melbourne at the Peter Doherty Institute for Infection and Immunity, Parkville, VIC 3010, Australia; Centre for Microbiology and Environmental Systems Science, University of Vienna, Vienna 1030, Austria; School of Biosciences, The University of Melbourne, Parkville, VIC 3010, Australia; School of Biosciences, The University of Melbourne, Parkville, VIC 3010, Australia; Australian Institute of Marine Science, Townsville, QLD 4810, Australia

**Keywords:** chlamydiae, alga, Symbiodiniaceae, genomics, coral

## Abstract

Chlamydiae are ubiquitous intracellular bacteria and infect a wide diversity of eukaryotes, including mammals. However, chlamydiae have never been reported to infect photosynthetic organisms. Here, we describe a novel chlamydial genus and species, *Candidatus Algichlamydia australiensis*, capable of infecting the photosynthetic dinoflagellate *Cladocopium* sp. (originally isolated from a scleractinian coral). *Algichlamydia australiensis* was confirmed to be intracellular by fluorescence *in situ* hybridization and confocal laser scanning microscopy and temporally stable at the population level by monitoring its relative abundance across four weeks of host growth. Using a combination of short- and long-read sequencing, we recovered a high-quality (completeness 91.73% and contamination 0.27%) metagenome-assembled genome of *A. australiensis*. Phylogenetic analyses show that this chlamydial taxon represents a new genus and species within the *Simkaniaceae* family. *Algichlamydia australiensis* possesses all the hallmark genes for chlamydiae–host interactions, including a complete type III secretion system. In addition, a type IV secretion system is encoded on a plasmid and has previously been observed for only three other chlamydial species. Twenty orthologous groups of genes are unique to *A. australiensis*, one of which is structurally similar to a protein known from *Cyanobacteria* and Archaeplastida involved in thylakoid biogenesis and maintenance, hinting at potential chlamydiae interactions with the chloroplasts of *Cladocopium* cells. Our study shows that chlamydiae infect dinoflagellate symbionts of cnidarians, the first photosynthetic organism reported to harbor chlamydiae, thereby expanding the breadth of chlamydial hosts and providing a new contribution to the discussion around the role of chlamydiae in the establishment of the primary plastid.

## Introduction

The *Chlamydiota* (also known as chlamydiae) is a phylum of obligate intracellular bacteria infecting eukaryotes [1, 2]. Despite their diversity, all known chlamydiae have a remarkably conserved biology; they are dependent on eukaryotic host cells for growth and survival, alternate between infectious extracellular elementary bodies and intracellular replicative reticulate bodies, and manipulate host cells through a type III secretion system (T3SS) [1]. Because of their intracellular lifestyle, all chlamydiae also possess reduced genomes (1–3 Mb) [3], and are not culturable *ex hospite*, making them notoriously difficult to study. Although chlamydiae are best known for infecting mammals, their host range is incredibly diverse and includes arthropods, amphibians, sponges, corals, and protists [2]. Thus far, however, there has been no record of photosynthetic organisms harboring chlamydiae. Intriguingly, genomic data suggest that numerous horizontal gene transfer events between chlamydiae and Archaeplastida (i.e. red algae, glaucophytes, and plants) have occurred, which were speculated to be remnants of ancestral interactions [4]. This led to the hypothesis that chlamydiae facilitated the establishment of ancestral *Cyanobacteria* as plastids [4, 5], though phylogenetic evidence for this hypothesis remains controversial [6].

Photosynthetic dinoflagellates of the *Symbiodiniaceae* family associate with a wide range of intra- and extracellular bacteria [7–11], some of which are beneficial for *Symbiodiniaceae* physiology and stress tolerance [12–17]. Even though they can be free-living, *Symbiodiniaceae* are widely known as endosymbionts of cnidarians, which they provide with organic carbon through photosynthate transfer [18]. Unlike Archaeplastida, photosynthetic dinoflagellates likely acquired their chloroplast from a secondary endosymbiotic event after the engulfment of an alga of the *Rhodophyta* taxon [19].

Chlamydiae sequences were recently detected in 16S rRNA gene amplicon sequencing of several *Symbiodiniaceae* laboratory cultures [7, 10, 15]. In a *Cladocopium* sp. culture (SCF049.01—see Supplementary text and Fig. S1 regarding its taxonomy), a single chlamydial amplicon sequence variant (ASV) made up >60% of the associated bacterial communities [7], but the nature of the association or whether *Cladocopium* is the true chlamydial host was not investigated. By combining amplicon sequencing, fluorescence microscopy, and genome sequencing and analysis, we prove that *Cladocopium* harbors chlamydial cells and provide a detailed description of an association between a chlamydial representative and a photosynthetic organism.

## Material and methods

### *Symbiodiniaceae* culture and maintenance

The *Symbiodiniaceae* culture SCF049.01 was used in this study (see Supplementary text for taxonomic considerations). It was initially isolated at the Australian Institute of Marine Science from the coral *Pocillopora damicornis* collected from Davies Reef (central Great Barrier Reef, Australia). *Symbiodiniaceae* cultures were maintained in 15 ml Daigo’s IMK medium (1×), prepared with filtered red sea salt water (fRSSW, 34 ppt salinity) in sterile 50 ml polypropylene culture flasks where media was changed fortnightly. These flasks were kept in a 12 h light:12 h dark incubator (50–60 μmol photons m^−2^ s^−1^ of photosynthetically active radiation) at 26°C.

### Fluorescence *in situ* hybridization on *Symbiodiniaceae* cells

Fluorescence *in situ* hybridization (FISH) was performed on *Symbiodiniaceae* cells as previously described [7] using the 16S rRNA-targeting, chlamydiae-specific probe Chls523 along with a competitor probe [20]. The antisense nonEUB probe [21] was also used as a negative control. Samples were observed on a Nikon Air Confocal Laser Scanning Microscope (CLSM). Additional details are available in the supplementary data.

### Transmission electron microscopy

For *Symbiodiniaceae* cell preparation for transmission electron microscopy (TEM), high-pressure freezing, freeze substitution, epoxy resin infiltration, sectioning, and observation were performed as previously described [22], with adaptations detailed in the supplementary data.

### Determination of chlamydial copy numbers by digital polymerase chain reaction (dPCR)

To determine chlamydial cell numbers, we performed digital dPCR on the supernatant and cellular fraction of six culture replicates. DNA was extracted from all samples using the DNeasy PowerSoil Pro Kit (Qiagen) according to the manufacturer’s instructions. Digital PCR was performed using the QIAcuity EvaGreen PCR kit (Qiagen) and the chlamydiae-specific primer pair Chl40F and Chl523R targeting the 16S rRNA gene on a QIAcuity One digital PCR device (Qiagen). The determined 16S rRNA gene copy numbers per milliliter correspond to chlamydial cell numbers per milliliter. In the cellular fraction, the chlamydial cell numbers per milliliter were normalized to the *Cladocopium* sp. cell counts. Additional details are available in the supplementary data.

### Growth phase and time series experiments (for 16S rRNA gene amplicon sequencing and flow cytometry)

To analyze *Cladocopium*-associated bacterial communities through time, the *Cladocopium* sp. culture was subcultured into three separate flasks at 1 × 10^5^ cells/ml in 100 ml 1X IMK media on Day 1. For the “growth phase” experiment, all three flasks were sampled on Days 1, 5, 8, 12, 15, 19, 22, 26, and 29, until the culture reached stationary phase (Fig. S2). All sampling was done between 14:00 and 16:00. On Days 8–9, during exponential phase, all three flasks were sampled across eight time points across the 2 days for the “time series” experiment (Fig. S2): 08:00 (Day 8), 12:00, 15:00, 18:00, 20:00, 00:00 (Day 9), 03:00, 06:00. Samples for Day 8 of the growth phase experiment, and for the 15:00 timepoint of the time series experiment were the same.

For each sampling day/time, cell density was obtained using a Countess II FL cell counter (Thermo Fisher scientific). Before each cell count, flasks were vigorously shaken to detach cells from the base of the flask and minimize any bias. Samples (100 μl) were taken from each of the three replicate flasks. Of this, two 10 μl samples were processed to obtain cell density for each flask. Cultures were then sampled as previously described [7] in order to separate “loosely associated,” “closely associated,” and “intracellular” bacteria from *Cladocopium* sp SCF049.01 cultures. Briefly, six replicates of 100 000 cells for each flask were filtered through a 5-μm strainer (pluriSelect, Germany), which retains *Cladocopium* cells (>5 μm), but not planktonic bacteria (<5 μm). Three replicates were washed with fRSSW to wash away bacteria that are not tightly attached to the cell surface. Filtrates represent the “loosely associated bacteria.” Filters were detached from the strainers and represent the “closely associated bacteria,” i.e. intracellular bacteria and those tightly attached to the surface. The other three replicates were washed with 6% sodium hypochlorite (v/v) to wash away all extracellular bacteria. Filters were detached from the strainers, and these represent the “intracellular bacteria.” Six filters (three for the growth phase experiment, three for the time series experiment) that only received fRSSW and six filters (three for the growth phase experiment, three for the time series experiment) that only received sodium hypochlorite (no algae) were also sampled as negative controls. All samples were snap-frozen and kept at −20°C until processing for 16S rRNA gene amplicon sequencing.

Additional samples were taken during the growth phase experiment for FISH and flow cytometry quantification of *Cladocopium* cells infected by chlamydiae, at Days 1, 8, 15, 22, and 29. Approximately 3 × 10^6^*Cladocopium* cells from each flask were sampled for each time point and fixed in 80% ethanol as previously described [7]. Because of the repeated centrifugations/washes during the FISH protocol, these samples are considered as the closely associated fraction. FISH and flow cytometry analyses were conducted as previously described [7], and additional details are available in the supplementary data.

### DNA extractions for amplicon sequencing

DNA extractions were performed using a salting-out method with modifications as previously described [23]. Extraction blanks (two to four) were included to account for potential contaminants introduced during the extraction process.

### Amplicon sequencing library preparation

For *Symbiodiniaceae* profiling, the ITS2 region was amplified using the primer pair Sym_Var_5.8S2 (5’ GTGACCTATGAACTCAGGAGTCGAATTGCAGAACTCCGTGAACC 3′) and Sym_Var_Rev (5’ CTGAGACTTGCACATCGCAGCCGGGTTCWCTTGTYTGACTTCATGC 3′) [24]. For 18S rRNA profiling, the primer pair 574*F (GTGACCTATGAACTCAGGAGTCCGGTAAYTCCAGCTCYV) [25] and 952R (CTGAGACTTGCACATCGCAGCTTGGCAAATGCTTTCGC) [26] was used. For bacterial communities, hypervariable regions V5–V6 of the 16S rRNA genes were amplified using the primer pair 784F (5′ GTGACCTATGAACTCAGGAGTCAGGATTAGATACCCTGGTA 3′) and 1061R (5′ CTGAGACTTGCACATCGCAGCCRRCACGAGCTGACGAC 3′). Underlined are the Illumina adapters attached to the primers. PCR amplification, library preparation, and sequencing were completed as previously described [27]. Four no-template PCRs were included per primer pair. Sequencing was performed on an MiSeq platform (Illumina) using v3 (2 × 300 bp) reagents at the Walter and Eliza Hall Institute (Melbourne, Australia).

### Amplicon sequencing data analyses

*Symbiodiniaceae* amplicon sequencing data (ITS2) were processed using the SymPortal analytical framework (symportal.org) [28]. Sequence information was submitted to the SymPortal remote database and underwent quality control including the removal of artefact and non-*Symbiodiniaceae* sequences. Relative abundances of ITS2 types were exported and plotted on GraphPad Prism 9.

18S rRNA gene amplicon sequencing data were processed using QIIME2 version 2021.8 [29]. Amplicon sequencing data were obtained as paired-end, demultiplexed files with primers and adapters attached. The cutadapt plugin [30] was used to remove primer and adapter sequences, with an error rate of 0.2. The quality of trimmed sequences was determined using the DADA2 plugin [31], which denoises, filters, dereplicates, detects chimeras, and merges paired-end reads. Reads with low quality (*Q*-score < 30) were removed. Taxonomy was assigned by training a naive Bayes classifier with the feature-classifier plugin [29], based on a 99% similarity to the 18S rRNA gene in the PR^2^ database v4.14.1 to match the primer pair used [32]. Metadata file, phylogenetic tree, and tables with amplicon sequence variant (ASV) taxonomic classifications and counts were exported, and relative abundances were plotted using GraphPad Prism 9.

16S rRNA gene amplicon sequencing data were processed using QIIME2 version 2021.8 [29]. Amplicon sequencing data were obtained as paired-end, demultiplexed files with primers and adapters attached. The cutadapt plugin [30] was used to remove primer and adapter sequences, with an error rate of 0.2. The quality of trimmed sequences was determined using the DADA2 plugin [31], which denoises, filters, dereplicates, detects chimeras, and merges paired-end reads. Reads with low quality (*Q*-score < 30) were removed. Taxonomy was assigned by training a naive Bayes classifier with the feature-classifier plugin [29], based on a 99% similarity to the V5–V6 region of the 16S rRNA gene in the SILVA 138 database to match the 784F/1061R primer pair used [33]. Mitochondria and chloroplast reads were filtered out. The metadata file, phylogenetic tree, and ASV tables were imported into Rstudio for analyses, using the phyloseq package [34]. At this stage of the analysis, datasets from the growth phase experiment and from the time series experiment were separated and analysed independently. Rare ASVs (percentage abundance lower than 1 × 10^−5^) were removed from the dataset. Samples with low read numbers were removed (four for the growth phase experiment: Day 5—Intracellular—Flask C, Day 15—Intracellular—Flask B, Day 22—Intracellular—Flask A, Day 26—Intracellular—Flask B; three for the time series experiment: 12:00—Loosely associated—Flask C, 0:00—Loosely associated—Flask A, 6:00—Loosely associated—Flask B). Sequencing statistics for all amplicon sequencing experiments can be found in Table S1. Contaminant ASVs, arising from kit reagents and sample manipulation, were identified manually based on their abundance in negative controls: any ASV that was five times more abundant in the mean abundance of filter blanks, extraction blanks, or no template PCRs compared to the mean of all *Cladocopium* sp SCF049.01 samples, and that represented at least 500 reads in all *Cladocopium* sp SCF049.01 samples, was considered a contaminant and removed from the dataset. Known contaminants (e.g. *Cutibacterium*) were also removed manually. For the growth phase experiments, 299 ASVs were identified as contaminants, accounting for 5.99% of *Cladocopium* sp SCF049.01 reads (Table S2A). For the time series experiment, 33 contaminants were identified, accounting for 9.88% of *Cladocopium* sp SCF049.01 reads (Table S2B). ASVs assigned to the chlamydiae were specifically targeted and plotted separately.

### Statistical analysis

Chlamydial abundance and the proportion of infected *Cladocopium* cells in the growth phase and time series experiments were analyzed and plotted using GraphPad Prism 9. Each experiment was analyzed independently. Within each experiment, the three different fractions (closely associated, intracellular, loosely associated) were analyzed independently. For each fraction, the effect of sampling time (day in the growth phase experiment, hour in the time series experiment) on chlamydial abundance and the proportion of infected *Cladocopium* cells was analyzed by performing a nonparametric Kruskal–Wallis test. Statistical tests were considered significant at α = 0.05, unless otherwise stated.

### Sample preparation and DNA extraction for long-read sequencing of *Cladocopium* microbial communities

For long-read sequencing, the supernatants of SCF049.01 cultures were sampled to minimize host DNA quantities. Two weeks after subculturing (*Cladocopium* density was around ~1 × 10^6^ cells/ml), 150 ml of supernatant were carefully removed, without disturbing the attached *Cladocopium* cells, to minimize *Cladocopium* contamination. The supernatant was subsequently filtered at 5 and 1.2 μm to remove *Cladocopium* cells and >1.2 μm-sized bacteria (chlamydiae are <1 μm). The filtered supernatant was centrifuged for 15 min at 3000 × *g* to pellet the bacteria. The supernatant was discarded and the bacterial pellet kept at −20°C. The bacterial pellet was lysed (lysozyme 100 mg/ml) and DNA was extracted with GenFind V3, according to manufacturer’s instructions for bacterial samples (Beckman Coulter). A ligation sequencing library was prepared (ONT SQK-NBD114–96) and the resultant library run on a MinION flow cell (FLO-MIN114) using a GridION device. Data were basecalled with Super-accurate basecalling in MinKNOW v23.07.05.

### Sample preparation and DNA extraction for short-read sequencing of *Cladocopium* microbial communities

For short-read sequencing, we employed a more comprehensive strategy to eliminate *Cladocopium* cells and DNA and maximize bacterial DNA yields. First, the “closely-associated” communities of around 5 × 10^6^*Cladocopium* cells were sampled as described above. Samples were then bead-beaten for 20 min at 30 Hz with 100 mg of sterile beads (400–600 nm) to open up the *Cladocopium* cells and stained with SYBR Green as previously described [12, 15]. Bacteria were then separated from *Cladocopium* cells and debris by fluorescence-activated cell sorting on an Aria III/FACS DiVa 9 software (BD Biosciences, Franklin Lakes, NJ) equipped with a 70 μm nozzle and run at 70 psi, as previously described [15]. Selection was based on size and high SYBR Green fluorescence. A total of 3.75 million events were obtained.

DNA was extracted using a HostZERO Microbial DNA Kit (Zymo) according to the manufacturer’s instructions. The final elution volume was 40 μl. A volume of 20 μl of purified DNA was concentrated using a DNA Concentrator Kit (abcam) according to the manufacturer’s instruction, with a final elution volume of 3 μl. The resulting DNA was amplified by multiple displacement amplification (MDA) using a REPLI-g Single Cell Kit (QIAGEN) following the manufacturer’s instructions. Following MDA at 30°C for 8 h, the DNA polymerase was inactivated, and amplified DNA was stored at −20°C. The sample was sequenced across two lanes of a NovaSeq 6000 SP 2 × 150 bp flowcell (Illumina, San Diego, CA) at the Ramaciotti Centre for Genomics (UNSW Sydney, Australia).

### Metagenomic analyses

Long-read sequence quality control was performed with NanoPlot v1.41.6 [35]. Raw sequences were trimmed with NanoFilt v2.8.0 [35], with parameters -q 10 -l 300 for quality and read length, respectively. Assembly was performed using Flye v2.9.2 [36] with two approaches: (i) using default genome mode for flye (flye --nano-raw reads) and (ii) metagenome mode (flye –meta –nano-raw reads). Assembly statistics from both approaches were compared and outputs from the metagenome mode were used for downstream analysis. Contig taxonomy was evaluated with CAT v5.3 [37] using default parameters and the non-redundant (NR) database, and with GTDB-Tk v2.3.0 using classify_wf [38]. Five contigs affiliated with the Chlamydiota phylum. All contigs were further binned with GraphMB v0.2.5 [39], and three additional contigs were binned along with the five contigs previously identified as chlamydial, resulting in an eight-contig bin.

The bin was subsequently polished using the short-read sequences. Short-read sequences were merged by read direction, and paired-end reads were quality-checked using FASTQC v0.11.5 (https://www.bioinformatics.babraham.ac.uk/projects/fastqc/). Reads were trimmed with trimmomatic v0.36 [40] with the following parameters: CROP:145, LEADING:30, HEADCROP:10, and MINLEN:120. Short reads were mapped to the bin obtained above using bowtie2 v2.4.2 [41] and Samtools v1.11 [42]. The bin was then polished with the mapped, high-quality short reads using Pilon v1.24 [43], twice successively. A hybrid assembly was attempted but did not result in a bin of higher quality.

Two contigs from the final bin were predicted to be circular. The content of one of the two contigs was not sufficient to function as a plasmid entity, and most chlamydiae only have a single plasmid [44], so we attempted to improve the contiguity of the circular contigs and obtain a single plasmid. Short reads were mapped to the two circular contigs using bowtie2 v2.4.2 [41] and Samtools v1.11 [42], and long reads were mapped to the two circular contigs using minimap2 v2.26 [45] and Samtools v1.11 [42]. A hybrid assembly was conducted with the mapped short and long reads and Unicycler v0.5.0 [46], yielding a single plasmid.

Bin quality and taxonomy were assessed using CheckM2 v1.0.2 [47] and GTDB-Tk v2.3.0 [38], respectively. Bin coverage was obtained using CoverM v0.6.1 (https://github.com/wwood/CoverM) using the “genome” option. Average amino acid identity (AAI) of the obtained bin was calculated with FastAAI (https://github.com/cruizperez/FastAAI) against 48 genomes of the *Simkaniaceae* and *Parasimkaniaceae* families.

### Phylogenetic analyses

For comparative and phylogenetic analyses, a dataset of high-quality chlamydial genomes based on Dharamshi *et al.* [48] was used. The chlamydial dataset was complemented by additional genomes available on GenBank/ENA/DDBJ on 29 May 2023. In general, only genomes with a completeness >70%, contamination <5% (both determined with CheckM2 v1.0.2 [47]), and average nucleotide identities <95% (determined with FastANI v1.33 [34]) were considered for the final dataset, which included 170 chlamydial genomes and 89 genomes from Planctomycetota and Verrucomicrobiota serving as outgroup (Table S3). To obtain the phylogenetic affiliation of Cla049, a set of 15 concatenated conserved nonsupervised orthologous groups (NOGs) was used (Table S4). These 15 NOGs are known to retrieve the same topology for chlamydial phylogeny as the application of larger protein sets and is now widely used for chlamydial phylogenies [48]. We remain careful with our conclusions regarding this analysis because of the relatively low number of genomes available. Proteins of the chlamydial and Planctomycetota-Verrucomicrobiota outgroup genomes belonging to the 15 NOGs were aligned with MAFFT v7.520 L-INS-i [49]. The resulting single protein alignments were subsequently trimmed with BMGE v2.0 [50] and concatenated. One chlamydial MAG with low completeness was excluded from phylogenetic analysis (Table S3). Maximum likelihood phylogeny was inferred with IQ-TREE v2.2.5 [51] using ModelFinder Plus (m MFP -mset LG,LG+C20 -mfreq “ “ -mrate G4,R4) [52] with 1000 ultrafast bootstrap replicates [53] and 1000 replicates of the SH-like approximate likelihood ratio test [54] under the LG + C20 + R4 model. The resulting phylogenetic tree was used as guide tree for maximum likelihood tree inference under the posterior site mean frequency (PMSF) model with 100 nonparametric bootstraps [55]. Phylogenetic trees were rooted using the outgroup and visualized with iTOL v6.8.1 [56].

### Genome annotation

Gene prediction was performed in Bakta v1.7.0 [57], KEGG-mapper Reconstruct [58], eggNOG-mapper v2.1.11 [59], and InterProScan v5.55 with Pfam domain annotations [60]. Pfam annotations were used to look for eukaryotic-like proteins (ankyrin-repeat domains, WD40 domains, tetratricopeptide repeat). Secondary metabolites were predicted using antiSMASH v7.0.0 [61]. The presence of specific genes of interest [virulence genes, T3SS, type IV secretion system (T4SS)] were investigated using BLASTp v.2.14.0 [62] and known sequences from the genomes of either *Simkania negevensis* [63] or *Chlamydia trachomatis* [64].

The genome annotation revealed the presence of five nucleotide transport protein (NTT) genes with one of them being a potential pseudogene separated into three CDSs (EFCAOE_00685, EFCAOE_00690, EFCAOE_00695). To decipher the potential transport capacities of this pseudogenized NTT, we merged the three single CDSs into one combined-CDS. All Cla049 NTTs were then added to a previously published dataset of NTTs [65] aligned with MAFFT v7.520 L-INS-i [49], and trimmed with BMGE v2.0 [50]. Maximum likelihood phylogeny of NTTs was inferred with IQ-TREE v2.2.5 [51] using ModelFinder Plus (-m MFP -mset LG,LG+C20 -mfreq “ “ -mrate G4,R4) [52] with 1000 ultrafast bootstrap replicates [53] and 1000 replicates of the SH-like approximate likelihood ratio test [54] under the LG + R4 model. The resulting phylogenetic was midpoint-rooted and visualized with iTOL v6.8.1 [56].

### Analysis of orthologous groups of proteins

For further comparative analysis of the chlamydial genomes, all encoded protein sequences were clustered into orthologous groups (OGs) with OrthoFinder v2.5.5 [66] under default parameters. OGs and their respective eggNOG annotations were merged in RStudio v2023.06.2 and analyzed. OGs present in the bin obtained in this study, but not present in any other chlamydial genome, were selected (this included all genes not classified in any OGs). Because most sequences did not show any similarity to known proteins in public databases, the structures of representative sequences of these OGs were predicted with AlphaFold v2.3.2 [67] and visualized with UCSF ChimeraX v1.6.1 [68]. The resulting best-ranked protein structure models were then searched with structure search against RCSB protein data bank (PDB) [69]. Only hits with global pLDDT >70 were considered in the results.

## Results and discussion

### Chlamydiae reside inside *Cladocopium* cells

**Figure 1:**
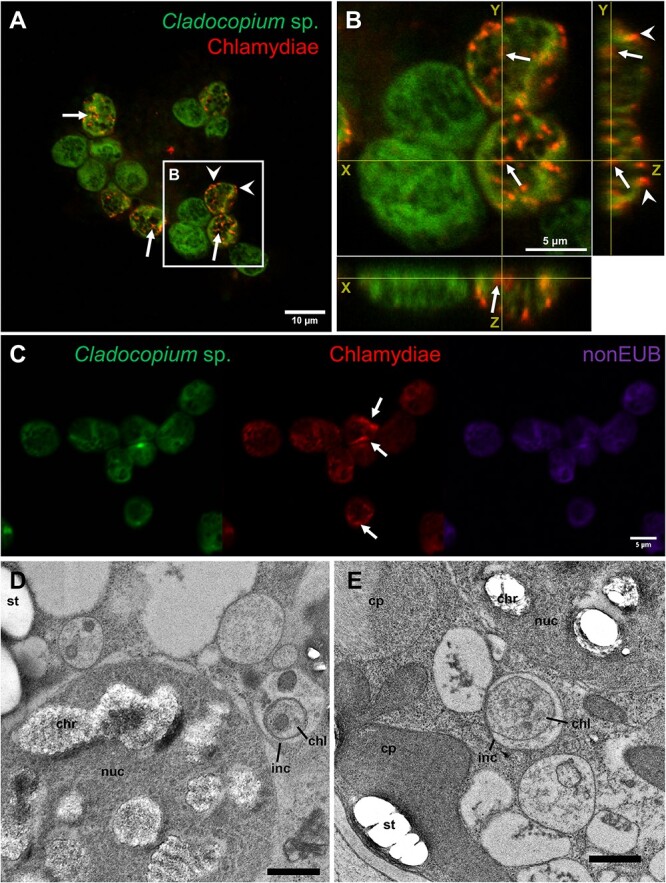
Chlamydiae infect *Cladocopium* sp. SCF049.01. (A–C) Chlamydiae located by FISH inside (white arrows) and on the cell wall (white arrowheads) of *Cladocopium* cells, observed by CLSM. Red: Chls523 probe (chlamydiae); green: *Cladocopium* autofluorescence; purple: nonEUB probe (antisense probe). (B) shows orthogonal projections of a Z-stack of four *Cladocopium* cells from (A), highlighting the presence of both intracellular and extracellular FISH signal. (D, E) Transmission electron micrographs showing chlamydial cells (chl) surrounded by inclusion membranes (inc) inside a *Symbiodiniaceae* cell. An overview image of the host cell containing the chlamydial cell in (E) is available as Fig. S4. Abbreviations: chl: chlamydial cell, inc: inclusion, nuc: nucleus, chr: chromosome, cp: chloroplast, st: starch granule. Scale bar: 500 nm.

To verify that the previously detected chlamydiae infect *Cladocopium* cells, rather than other protists potentially present in the culture, we conducted 18S rRNA gene amplicon sequencing. All reads from three replicate culture flasks were assigned to *Symbiodiniaceae* (Fig. S3), strongly suggesting that there were no other eukaryotes and thus, no other chlamydial hosts, in the culture. Additionally, FISH and confocal laser scanning microscopy (CLSM) using a chlamydiae-specific probe clearly showed whole bacterial cells fluorescing inside *Cladocopium* cells, as well as on their cell wall (Fig. 1A–C). Chlamydiae are usually encased in large host-derived inclusions inside host cells [70], though they can sometimes reside in single-cell inclusions [71] or in the cytoplasm [72]. Transmission electron microscopy confirmed the presence of chlamydial cells (~400–600 nm in diameter) inside single-cell inclusions within *Symbiodiniaceae* (Fig. 1D and E and S4). This is similar to previously observed single-cell inclusions of *Protochlamydia amoebophila* in the amoeba *Acanthamoeba castellanii* [71], though chlamydial density in *Cladocopium* cells appears much lower.

**Figure 2:**
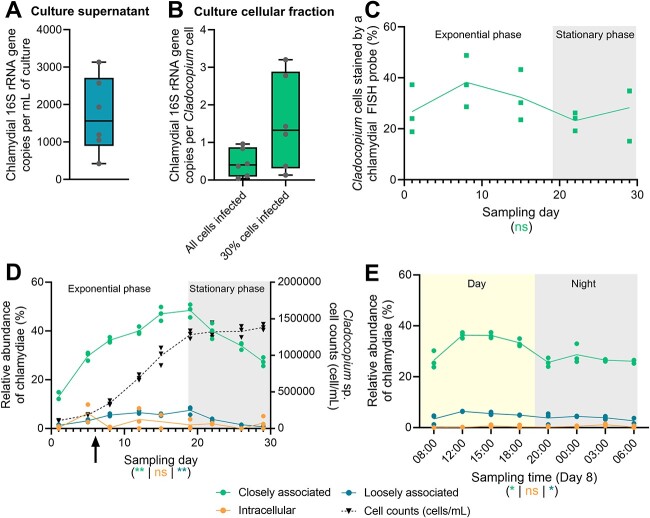
Chlamydial infection of *Cladocopium* sp. is temporally stable. (A, B) Chlamydial abundance in culture supernatant (A) or cellular fraction (B), measured by digital PCR. For the cellular fraction (B), chlamydial gene copy data were normalized to either total *Cladocopium* cell numbers (i.e. every *Cladocopium* cell is infected) or 30% of *Cladocopium* cell numbers [i.e. only 30% of cells harbor chlamydiae; see (C)]. Boxes represent first quartile to third quartile for six independent replicates (also shown individually), the middle lines represent the medians, and the whiskers represent the minimum and maximum values. (C) Proportion of *Cladocopium* sp. cells stained by FISH with the chlamydiae-specific probe Chls523, as analyzed by flow cytometry. Each point represents one of three replicate flasks, with lines connecting the means. Gray-shaded areas represent dark time, while white areas represent light time. The effect of sampling day is indicated under the plot with its corresponding color, based on a Kruskal–Wallis test (ns: *P* > .05). (D, E) Relative abundance of chlamydial ASVs in the *Cladocopium* culture across 29 days (D, growth phase experiment) or 24 h (E, time series experiment, performed on Day 8 of the growth phase experiment—see black arrow in D), determined by 16S rRNA gene amplicon sequencing (see Fig. S2 for experimental design). Bacterial community profiling was performed in three fractions as previously described [7]: loosely associated bacteria (planktonic bacteria; blue), closely associated (intracellular and tightly attached to the cell wall; green) and intracellular bacteria (orange). *Cladocopium* cell counts across the growth phase experiments are also provided (dashed black line, D). Each point represents one of three replicate flasks, with lines connecting the means. For each fraction, the effect of sampling day or time on chlamydial relative abundance is indicated under the plot with its corresponding color, based on a Kruskal–Wallis test. ns: *P* > .05; *: *P* ≤ .05; **: *P* ≤ .01. Single replicate values are available in Table S5.

Absolute quantification of chlamydiae through digital PCR (dPCR) revealed that there were an average of 1716 chlamydial cells/ml of culture supernatant (Fig. 2A) and that each *Cladocopium* cell was infected by an average 0.46 chlamydial cells (Fig. 2B) suggesting the presence of the common chlamydial developmental cycle with extracellular elementary bodies and intracellular replicative bodies. Because not all *Cladocopium* cells harbored chlamydiae (Fig. 1A–C), we measured the number of *Cladocopium* cells stained by FISH (both intracellularly and attached to the cell wall) by flow cytometry across a host growth cycle (once a week for 4 weeks, Fig. S2). This showed that an average of 30% of cells were infected by chlamydiae across four time points (Fig. 2C). Therefore, using our previous chlamydial absolute quantification, each infected *Cladocopium* cell (i.e. 30% of the total number of cells) harbors an average of 1.52 chlamydial cells (Fig. 2B).

### *Cladocopium*-chlamydiae infection is temporally stable

The temporal stability of the association was assessed at the population level by characterizing the bacterial communities throughout a host growth cycle (twice a week for 4 weeks) and across a single day (eight time points across 24 h, including four during the day and four at night) (Fig. S2 and Table S1). Bacterial communities were sampled in three fractions as previously described [7]: loosely associated (planktonic bacteria), closely associated (intracellular and tightly attached to the cell wall), and intracellular. In both experiments, a single chlamydial ASV made up the majority of the reads (Table S5). Chlamydial relative abundance was highest in the closely associated fraction, where it increased from 13% to 48% during host exponential phase and decreased once stationary phase was reached (Figs 2D and S5A and Table S5A). The same trend was observed in the loosely associated fraction, with a peak at 7.5% in relative abundance during host exponential phase. These findings suggest chlamydiae may replicate or be transmitted more easily when their host cells are dividing. The relative abundance of closely associated chlamydiae was higher during the day (33.1% across the four time points) than at night (26.7%) (Fig. 2E and S5B and Table S5B). This may be due to the lack of host photosynthesis at night, resulting in lower amounts of adenosine triphosphate (ATP) available for chlamydial survival and replication. Chlamydial relative abundance in the intracellular fraction was very low (<5%) in both experiments, suggesting elementary bodies might be more abundant than replicative bodies in this culture. Alternatively, it might be the result of a technical bias from the sodium hypochlorite treatment during sample fractionation, which may affect bacterial DNA even inside *Symbiodiniaceae* cells and introduce biases in the measured relative abundances. Thus, the results from the intracellular fraction are to be treated carefully.

### *Cladocopium*-associated chlamydiae belong to an undescribed *Simkaniaceae* genus

Using a combination of long- and short-read sequencing, we recovered a metagenome-assembled genome (MAG) of the *Cladocopium*-associated chlamydiae (Cla049; Table 1). The Cla049 MAG is comprised of 1 799 045 bp across seven contigs and estimated to be 91.73% complete. The MAG also includes one 87 402 bp plasmid (pAa). The last common ancestor of all chlamydiae likely possessed a plasmid, which was lost and/or integrated into the chromosome of some chlamydial lineages, but conserved in others [44, 63]. Cla049’s plasmid possesses key genes present on other chlamydial plasmids, including *pgp1* (a replicative DNA helicase) and *pgp2* (a virulence protein) (Table S6). Other genes typically present on chlamydial plasmids, such as *praA*/*pgp5* (a chromosome partitioning protein) and *pgp6* (involved in host cell response mediation), are on noncircular contigs and therefore may have been integrated into the genome. Cla049 encodes 11 transposases, including two on its plasmid, hinting at a potential for gene flow between the plasmid and chromosome that may explain the presence of plasmid genes on noncircular contigs.

**Table 1:** Summary statistics from the *Candidatus Algichlamydia australiensis* genome (Cla049 MAG).

**Genome**	**Cla049**
**Size (bp)**	1 799 045
**Long-read coverage (X)**	10
**Short-read coverage (X)**	2253
**Completeness (%)**	91.73
**Contamination (%)**	0.27
**Estimated complete size (Mb)**	2.0
**G + C content (%)**	42.1
**N50**	497 235
**Number of contigs**	7
**Number of coding sequences (CDSs)**	1644
**Number of ribosomal RNA operons**	1
**Number of transfer RNAs**	21
**Plasmids**	1 (pAa)
**Plasmid size (bp)**	87 402

**Figure 3:**
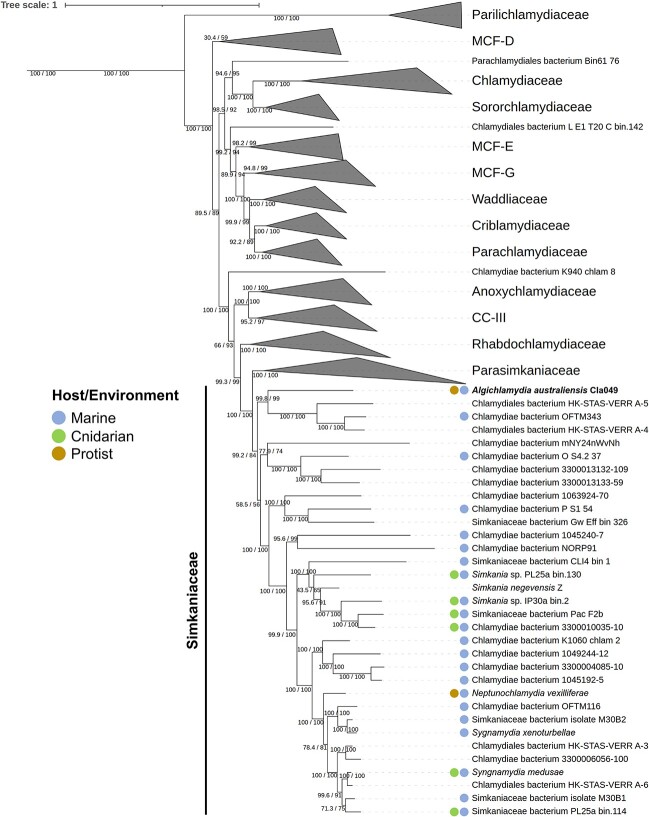
The chlamydial symbiont *Candidatus* Algichlamydia australiensis (Cla049 MAG) belongs to an undescribed, deep-branching *Simkaniaceae* genus. Chlamydial maximum likelihood phylogeny based on 15 conserved gene markers (Table S4) in 169 chlamydial genomes (Table S3). Confidence values based on 1000 ultrafast bootstrap replicates and 1000 replicates of the SH-like approximate likelihood ratio test are provided. Scale bar represents 1 nucleotide substitution per site. This tree was calculated using IQ-TREE 2 [51] with ModelFinder Plus under the LG + C20 + R4 model [52]; an additional tree calculated under the posterior mean site frequency (PMSF) model [55] using this tree as seed confirmed the phylogeny and is available in Fig. S6. MCF: metagenomic chlamydial family; CC-III: chlamydiae clade III.

To assess the taxonomic placement of the Cla049 MAG, we constructed two maximum likelihood phylogenetic trees based on 15 conserved marker genes (Table S4) and 169 chlamydial genomes (Table S3), calculated with IQ-TREE using different models (Figs 3 and S6). Cla049 represents an undescribed, deep-branching genus within the *Simkaniaceae* family. Cla049 is most closely related to a group of three MAGs, including two isolated from activated sludge in Hong Kong (HK-STAS-VERR_A-4 and HK-STAS-VERR_A-5), and one from a marine microbial biofilm in Norway (OFTM343). Average AAI is ~40–47% with all *Simkaniaceae* and *Parasimkaniaceae* genomes (Table S7), supporting the placement of Cla049 into a novel genus and species, which we propose to name *Candidatus* Algichlamydia australiensis gen. nov. sp. nov. (*A. australiensis* Cla049 hereafter). The name has been registered through SeqCode [73].

### *Algichlamydia australiensis* Cla049 has a reduced metabolic potential

The metabolic potential of *A. australiensis* is heavily reduced (Tables 2 and S8), lacking pathways for the synthesis of nucleotides, vitamins, and most amino acids (only genes for glutamate, aspartate, lysine, and alanine biosynthesis were found, as well as glycine–serine interconversion). The *A. australiensis* genome contains the genes necessary for glycolysis, the tricarboxylic acid cycle, pyruvate oxidation, the pentose phosphate pathway, and glycogenesis. *Algichlamydia australiensis* also possesses a complete shikimate pathway, as well as a complete menaquinone biosynthesis pathway, a cofactor that promotes growth in *C. trachomatis* [74]. Finally, the genome was predicted to produce two secondary metabolites, most closely related to nostovalerolactone (Table S9), a putative transcriptional regulator [75]. These metabolic abilities, or lack thereof, are similar to other chlamydiae [48, 76] and suggest that *A. australiensis* acquires most metabolites and energy from its *Cladocopium* host.

**Table 2:** List of complete and incomplete metabolic pathways in *Algichlamydia australiensis* Cla049.

**Category**	**Pathways > 80% complete**	**Incomplete pathways**
**Carbon metabolism**	Glycolysis (Embden–Meyerhof pathway)	Entner–Doudoroff pathway
	Pentose phosphate pathway	PRPP biosynthesis
	Citrate cycle (TCA cycle)	
	Pyruvate oxidation	
	Glycogen biosynthesis	
**Nitrogen metabolism**		Denitrification
		Dissimilatory nitrate reduction
		Assimilatory nitrate reduction
**Nucleotide biosynthesis**		*De novo* purine biosynthesis
		*De novo* pyrimidine biosynthesis
**Amino acid biosynthesis**	Glycine–serine interconversion	Asparagine biosynthesis
	Aspartate biosynthesis	Glutamine biosynthesis
	Alanine biosynthesis	Serine biosynthesis
	Glutamate biosynthesis	Threonine biosynthesis
	Lysine biosynthesis	Ectoine biosynthesis
	Shikimate pathway	Cysteine biosynthesis
		Methionine biosynthesis
		Valine/isoleucine biosynthesis
		Leucine biosynthesis
		Arginine biosynthesis
		Proline biosynthesis
		Histidine biosynthesis
		Tryptophan biosynthesis
		Phenylalanine biosynthesis
		Tyrosine biosynthesis
		Spermidine biosynthesis
		Putrescine biosynthesis
		Glutathione biosynthesis
**Cofactor biosynthesis**	Menaquinone biosynthesis	Thiamine biosynthesis
		Riboflavin biosynthesis
		Pyridoxal biosynthesis
		Pantothenate biosynthesis
		Pimeloyl-ACP biosynthesis
		Cobalamin biosynthesis
		Biotin biosynthesis
		Tetrahydrofolate biosynthesis
		Siroheme biosynthesis
		Heme biosynthesis
		NAD biosynthesis
		Coenzyme A biosynthesis
		Ubiquinone biosynthesis

*Algichlamydia australiensis* was predicted to encode five nucleotide transport proteins (NTTs), which were all most closely related to the four NTTs of *S. negevensis*, the *Simkaniaceae*-type species (Fig. S7). NTTs are critical for chlamydiae to import essential nutrients, including ATP, from their host cell [77, 78]. It is worth noting that plastid ATP/ADP antiporters in photosynthetic eukaryotes are derived from chlamydiae [79]. In *S. negevensis*, *Sn*NTT1 is an ATP/ADP antiporter, *Sn*NTT2 is a guanine nucleotide/ATP/H^+^ symporter, *Sn*NTT3 acts as an RNA nucleotide antiporter, and no substrate was identified for *Sn*NTT4 [78]. However, *Sn*NTT1’s putative ortholog in *A. australiensis* Cla049 was split into three CDSs (EFCAOE_00685/00690/00695), with a STOP codon at the end of EFCAOE_00690 and a STOP codon and a frameshift at the end of EFCAOE_00695, and is thus presumably not functional, drastically diminishing the potential for ATP import and consequently energy parasitism. Nonetheless, *A. australiensis* Cla049 appears to possess two orthologs of *Sn*NTT3 (Fig. S7; EFCAOE_00240 and EFCAOE_06465), which can import ATP in exchange for other nucleotides and may therefore compensate for the hypothetical nonfunctionality of NTT1. Experimental analyses are needed to determine the true substrates of *A. australiensis* Cla049’s NTTs. Alternatively, because *Cladocopium* sp. is photosynthetic, it may have lower intracellular ATP and higher intracellular glucose than heterotrophic host cells, and *A. australiensis* Cla049 may rely more on glycolysis than ATP parasitism.

### The chromosome and plasmid of *Algichlamydia australiensis* Cla049 encode a type III secretion system and type IV secretion system, respectively

**Figure 4:**
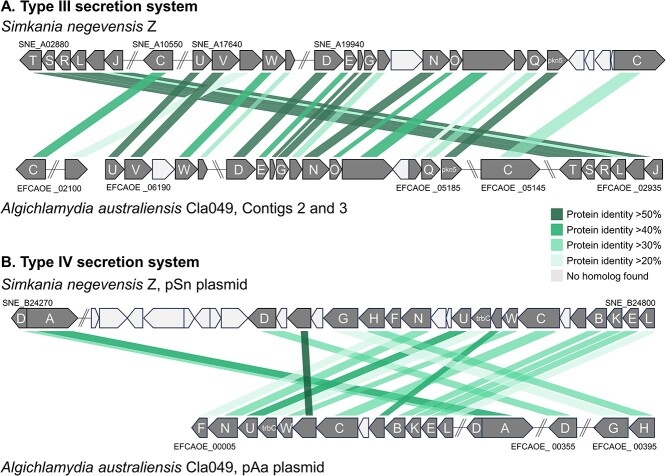
Completeness, similarity, and synteny of the genomic regions encoding the T3SS (A, *sct* genes) and T4SS (B, *tra* genes) in *Algichlamydia australiensis* Cla049, compared with *Simkania negevensis* Z. Synteny is shown by lines joining the two genomes; protein identity is color-coded.

*Algichlamydia australiensis* Cla049 encodes most chlamydial hallmark virulence-associated genes that are present in other chlamydiae (Table S10) [76]. This includes adhesins, T3SS effectors, Ser/Thr kinases (for host cell modulation), and developmental regulators. We also found 17 genes encoding putative eukaryotic-like repeat proteins (12 ankyrin repeat proteins, 1 WD40 repeat protein, and 4 tetratricopeptide repeat proteins), which are putative mediators of host–bacteria interactions and also abundant in other chlamydiae [63]. Finally, *A. australiensis* Cla049 encodes a complete T3SS and T4SS (Fig. 4), the latter being encoded on the plasmid. Both secretion systems show high synteny with the corresponding coding regions of the chromosome and plasmid, respectively, of *S. negevensis* (Fig. 4). The chlamydial T3SS is highly conserved and allows for the translocation of effectors into eukaryotic host cells [80]. The T4SS has only been reported in *Simkaniaceae* and *Parachlamydiaceae*, and its role remains unclear, though it may be involved in plasmid propagation [44, 63, 81]. The chlamydial T4SS is plasmid-encoded in only three other known chlamydial species, *S. negevensis*, *Protochlamydia naegleriophila*, and *Rubidus massiliensis*, and T4SS-encoding genes have been integrated in the chromosome of many *Parachlamydiaceae* and *Simkaniaceae* species [44, 63]. It was hypothesized that a T4SS of alphaproteobacterial origin was integrated into the plasmid of the *Parachlamydiaceae* ancestor and subsequently acquired by the plasmid of the *Simkaniaceae* ancestor [44]. The presence of a plasmid-encoded T4SS in two distinct *Simkaniaceae* genera, *Simkania* and *Algichlamydia*, supports this hypothesis.

### Unique genes in *A. australiensis* Cla049 may impact host–symbiont interactions

Because *A. australiensis* Cla049 is the first confirmed chlamydial symbiont of a photosynthetic organism, we asked whether it possessed any unique genes, not present in other known chlamydiae, which may mediate interactions with a photosynthetic host. We found 20 orthologous groups (OGs) that were specific to *A. australiensis* Cla049 (Table S11), as well as 189 genes that were not classified into any OG (Table S12). Only one OG (OG0007644) was reliably annotated and was predicted to encode a lanthionine synthetase. Lanthionine is a major component of lantibiotics, a class of antimicrobial compounds [82]. Some chlamydiae defend their hosts against pathogens; for exampl e, *Parachlamydia acanthamoebae* protects its amoeba host against viruses [83]. Lantibiotics may protect *Symbiodiniaceae* from bacterial pathogens; alternatively, they may help reduce competition from other bacteria within *Symbiodiniaceae* cultures and facilitate chlamydial growth. Symbiodiniaceae

Other OGs and unclassified genes were predicted to encode hypothetical proteins without any known motifs. Thus, we predicted the structure of one representative sequence per OG using AlphaFold and queried the RCSB Protein Data Bank to identify structurally similar proteins. Only 29 genes, including 4 out of the 20 OGs, resulted in reliable structural predictions (pLDDT >70; Fig. S8 and Tables S11 and S12). Among them, one gene (EFCAOE_07285) had two particularly relevant close hits: (i) a putative ankyrin repeat protein, which may be involved in host–chlamydiae interactions, and (ii) a vesicle-inducing protein in plastids 1 (VIPP1), a protein involved in the biogenesis and integrity of thylakoid membranes of plants, algae, and cyanobacteria [84]. In *Arabidopsis thaliana*, VIPP1 knockdown results in thylakoid swelling under high light [85], and VIPP1 overexpression improves postheat stress recovery [86]. Therefore, EFCAOE_07285 may mediate interactions between *A. australiensis* Cla049 and *Cladocopium*’s thylakoids and impact the photophysiology of *Cladocopium* cells.

### *In hospite Symbiodiniaceae* may not harbor *Algichlamydia australiensis* Cla049

Our previous study of *Symbiodiniaceae*-associated bacteria showed that ASVs at least 99% identical to *A. australiensis* Cla049 also infects cultures of *Gerakladium* sp. G3, *Breviolum minutum*, *Durusdinium trenchii*, and *Fugacium* sp. F5.1, though at much lower abundances than in *Cladocopium* sp. SCF049.01 (Table 3) [7]. Because these *Symbiodiniaceae* cultures are themselves symbionts of cnidarians, we asked whether *A. australiensis* Cla049 infects *in hospite Symbiodiniaceae* (within cnidarians) or cnidarians themselves that are known to be chlamydial hosts [2, 87–89]. Analysis of a recent dataset of 12 009 cnidarian 16S rRNA gene amplicon sequencing samples from 186 studies [90] showed that only five samples (each from a different study) harbored ASVs at least 99% identical to the 16S rRNA sequence of *A. australiensis* Cla049 (Table 3). The five samples were from four different genera of scleractinian corals, and the relative abundance of *A. australiensis* Cla049 varied between 0.01% and 0.31% (Table 3). This may be underestimated because of known biases from common universal primers against chlamydiae (e.g. 515F/806R of the V4 region, or 1391R of the V8 region [91]), as well the low numbers of *A. australiensis* Cla049 in *Cladocopium* cells, which may be missed if the sequencing depth is too low. Nonetheless, this low abundance and prevalence also suggest that *A. australiensis* Cla049 is not a common symbiont of *in hospite Symbiodiniaceae* or cnidarians, and perhaps does not interact with cnidarian hosts at all. *A. australiensis* Cla049 may not be able to bypass the cnidarian immune system, therefore not having direct access to *in hospite Symbiodiniaceae*. Additionally, *Symbiodiniaceae* cell division is much slower *in hospite* compared to free-living *Symbiodiniaceae* [92]. This may limit chlamydial growth and transmission opportunities among *in hospite Symbiodiniaceae*.

**Table 3:** Relative abundance of *Algichlamydia australiensis* Cla049 in *Symbiodiniaceae* (A) and cnidarian (B) 16S rRNA gene metabarcoding samples. The *Symbiodiniaceae* data were obtained from a previous study that analyzed bacterial community composition in 11 *Symbiodiniaceae* cultures [7]. The cnidarian data were obtained from a recent dataset combining 186 cnidarian microbiome studies and 12 009 cnidarian samples [90].

***SYMBIODINIACEAE***								
**Sample ID**	**Relative abundance of *Algichlamydia australiense* Cla49 (%)**	**Host species**	**Culture ID**	**Original cnidarian host**	**Original waterbody**	**Collection**	**Fraction of the bacterial community**	**Publication**
SRR12524345	0.65	*Gerakladium* sp. (G3)	SCF097.01	*Diploastrea heliopora*	Culture	Lab	Intracellular	[7]
SRR12524358	1.305	*Fugacium* sp. (F5.1)	SCF092.01	*Pocillopora damicornis*	Culture	Lab	Intracellular	
SRR12524393	0.455	*Durusdinium trenchii*	SCF082	*Acropora muricata*	Culture	Lab	Intracellular	
SRR12524394	0.10	*Durusdinium trenchii*	SCF082	*Acropora muricata*	Culture	Lab	Intracellular	
SRR12524424	3.16	*Breviolum minutum*	MMSF01	*Exaiptasia diaphana*	Culture	Lab	Closely associated	
SRR12524426	0.02	*Breviolum minutum*	MMSF01	*Exaiptasia diaphana*	Culture	Lab	Closely associated	
SRR12524429	0.01	*Breviolum minutum*	MMSF01	*Exaiptasia diaphana*	Culture	Lab	Loosely associated	
SRR12524431	0.06	*Breviolum minutum*	MMSF01	*Exaiptasia diaphana*	Culture	Lab	Intracellular	
SRR12524433	0.03	*Breviolum minutum*	MMSF01	*Exaiptasia diaphana*	Culture	Lab	Intracellular	
**CORAL**								
**Sample ID**	**Relative abundance of *Algichlamydia australiense Cla49* (%)**	**Host species**	**Lifestage**	**Host tissue**	**Original_Waterbody**	**Collection**	**Condition**	**Publication**
SRR1772480	0.31	*Acropora muricata*	Adult	Tissue	China Sea	Lab	Healthy	[93]
SRR9264720	0.01	*Montipora stellata*	Adult	Tissue	Singapore Strait	Lab	Healthy	[94]
ERR2816723	0.04	*Astrea curta*	Adult	Skeleton	Tasman Sea	Field	Healthy	[95]
SRR12284493	0.02	*Pocillopora* sp.	Adult	Tissue	Indian Ocean	Lab	Healthy	[96]
SRR12773401	0.01	*P. damicornis*	Adult	Tissue	China Sea	Lab	Stressed	[97]

## Conclusion

We provided a genomic characterization of a member of the chlamydiae that infects cultures of the dinoflagellate *Cladocopium* sp. SCF049.01. Although *Symbiodiniaceae* have been shown to feed on bacteria in some cases [98], it is unlikely to be the case here for the following reasons: (i) chlamydiae are known to escape host phagocytosis once internalized and avoid digestion [1, 99], and (ii) high relative abundances of *Simkaniaceae* in this *Cladocopium* sp. culture were detected as far back as 2019 [7], in 2021 (sampling for the growth phase and time series experiments, as well as for the short-read metagenomic sequencing), in 2022 (sampling for the flow cytometry experiment), and in 2023 (sampling for the digital PCR and long-read metagenomic sequencing), confirming the long-term stability of this association. Further investigation is required to determine the potential influence of *A. australiensis* on their *Cladocopium* sp*.* host. Whether mutualistic (e.g. by protecting from other pathogens) or pathogenic, this infection would likely influence the survival and proliferation of free-living *Symbiodiniaceae*. By increasing or decreasing the available pool of free-living *Symbiodiniaceae*, this chlamydial infection may, in turn, affect the colonization of cnidarian hosts and the health of coral reefs—despite our findings that *A. australiensis* does not seem to be present in cnidarian holobionts.

As far as we know, a chlamydial infection in a photosynthetic organism has never been described before [2]. Nonetheless, chlamydial 16S rRNA gene reads were recently detected in cultures of the green alga *Ostreobium* sp. [100, 101], and chlamydial MAGs were retrieved from cultures of the green alga *Amoebophrya* sp. and the kelp *Saccharina japonica* [102]. However, in all these examples, it was not established whether the photosynthetic organism or associated protists were the chlamydial host. Thus, additional studies are required to fully appreciate the potential breadth of photosynthetic hosts of chlamydiae, which would provide valuable insight into the evolutionary history of chlamydiae. This example of a chlamydia infecting a photosynthetic host cell also sheds new light on a hypothesis about a possible contribution of chlamydiae to the establishment of cyanobacterial symbionts during the evolution of the first photosynthetic eukaryotes [4–6].

## Supplementary Material

supplementary_algichlamydia_revision3_wrae139

Table_S2-contaminants_wrae139

Table_S3-genome_dataset_wrae139

Table_S5-Simkaniaceae_abundance_in_GP_and_TS_wrae139

Table_S7-AAI_wrae139

Table_S8-bakta_eggnog_wrae139

Table_S10-virulence_gene_annotation-clean_wrae139

Table_S11-OGs_wrae139

Table_S12-cla049_singletons_wrae139

## Data Availability

All data needed to evaluate the conclusions in the paper are present in the paper and/or the Supplementary Materials. Raw data are available under the following NCBI BioProject IDs: PRJNA935927 (MiSeq raw data for Growth phase and Time series experiments); PRJNA1018284: (MiSeq raw data for 18S rRNA and ITS2 gene amplicon sequencing of *Cladocopium* sp. SCF049.01); PRJNA1036848 (raw NovaSeq and minION metagenome sequencing, and the Cla049 MAG [JAXCHI000000000]). Individual sequences are available under the following NCBI GenBank IDs: *Cladocopium* sp. SCF049.01 ITS2 gene: OR564117; *Cladocopium* sp. SCF049.01 LSU gene OR564118; *Cladocopium* sp. SCF049.01 cp23S gene: OR575593; *Cladocopium* sp. SCF049.01 *cob* gene: OR568575; *Cladocopium* sp. SCF049.01 *cox1* gene: OR568576; *Algichlamydia australiensis* Cla049 16S rRNA gene: OR835247.

## References

[1] Horn M . Chlamydiae as symbionts in eukaryotes. *Ann Rev Microbiol* 2008;62:113–31. 10.1146/annurev.micro.62.081307.162818 18473699

[2] Collingro A , Köstlbacher S, Horn M. Chlamydiae in the environment. *Trends Microbiol* 2020;28:877–88. 10.1016/j.tim.2020.05.020 32591108

[3] Dharamshi JE , Köstlbacher S, Schön ME et al. Gene gain facilitated endosymbiotic evolution of Chlamydiae. *Nat Microbiol* 2023;8:40–54. 10.1038/s41564-022-01284-9 36604515 PMC9816063

[4] Huang J , Gogarten JP. Did an ancient chlamydial endosymbiosis facilitate the establishment of primary plastids? *Genome Biol* 2007;8:R99. 10.1186/gb-2007-8-6-r99 17547748 PMC2394758

[5] Cenci U , Bhattacharya D, Weber APM et al. Biotic host–pathogen interactions As Major drivers of plastid endosymbiosis. *Trends Plant Sci* 2017;22:316–28. 10.1016/j.tplants.2016.12.007 28089380

[6] Domman D , Horn M, Embley TM et al. Plastid establishment did not require a chlamydial partner. *Nat Commun* 2015;6:6421. 10.1038/ncomms7421 25758953 PMC4374161

[7] Maire J , Girvan SK, Barkla SE et al. Intracellular bacteria are common and taxonomically diverse in cultured and in hospite algal endosymbionts of coral reefs. *ISME J* 2021;15:2028–42. 10.1038/s41396-021-00902-4 33558689 PMC8245515

[8] Lawson CA , Raina J-B, Kahlke T et al. Defining the core microbiome of the symbiotic dinoflagellate, *Symbiodinium*. *Environ Microbiol Rep* 2018;10:7–11. 10.1111/1758-2229.12599 29124895

[9] Maire J , Philip GK, Livingston J et al. Functional potential and evolutionary response to long-term heat selection of bacterial associates of coral photosymbionts. *mSystems* 2023;8:e00860–23. 10.1128/msystems.00860-23 37909753 PMC10746172

[10] Buerger P , Vanstone RT, Maire J et al. Long-term heat selection of the coral endosymbiont Cladocopium C1acro (Symbiodiniaceae) stabilizes associated bacterial communities. *Int J Mol Sci* 2022;23:4913. 10.3390/ijms23094913 35563303 PMC9101544

[11] Maire J , Blackall LL, van Oppen MJH. Intracellular bacterial symbionts in corals: challenges and future directions. *Microorganisms* 2021;9:2209. 10.3390/microorganisms9112209 34835335 PMC8619543

[12] Heric K , Maire J, Deore P et al. Inoculation with Roseovarius increases thermal tolerance of the coral photosymbiont, Breviolum minutum. *Front Ecol Evol* 2023;11:1079271. 10.3389/fevo.2023.1079271

[13] Motone K , Takagi T, Aburaya S et al. A Zeaxanthin-producing bacterium isolated from the algal phycosphere protects coral endosymbionts from environmental stress. *MBio* 2020;11:e01019–9. 10.1128/mBio.01019-19 31964724 PMC6974559

[14] Matthews JL , Khalil A, Siboni N et al. Coral endosymbiont growth is enhanced by metabolic interactions with bacteria. *Nat Commun* 2023;14:6864. 10.1038/s41467-023-42663-y 37891154 PMC10611727

[15] Maire J , Deore P, Jameson VJ et al. Assessing the contribution of bacteria to the heat tolerance of experimentally evolved coral photosymbionts. *Environ Microbiol* 2023;25:3298–318. 10.1111/1462-2920.16521 37849020

[16] Matthews JL , Hoch L, Raina J-B et al. Symbiodiniaceae photophysiology and stress resilience is enhanced by microbial associations. *Sci Rep* 2023;13:20724. 10.1038/s41598-023-48020-9 38007500 PMC10676399

[17] Maire J , van Oppen MJH. A role for bacterial experimental evolution in coral bleaching mitigation? *Trends Microbiol* 2022;30:217–28. 10.1016/j.tim.2021.07.006 34429226

[18] Trench RK . Microalgal-invertebrate symbioses: a review. *Endocytobiosis Cell Res* 1993;9:135.

[19] Ponce-Toledo RI , López-García P, Moreira D. Horizontal and endosymbiotic gene transfer in early plastid evolution. *New Phytol* 2019;224:618–24. 10.1111/nph.15965 31135958 PMC6759420

[20] Poppert S , Essig A, Marre R et al. Detection and differentiation of chlamydiae by fluorescence in situ hybridization. *Appl Environ Microbiol* 2002;68:4081–9. 10.1128/AEM.68.8.4081-4089.2002 12147510 PMC124059

[21] Wallner G , Amann R, Beisker W. Optimizing fluorescent in situ hybridization with rRNA-targeted oligonucleotide probes for flow cytometric identification of microorganisms. *Cytometry* 1993;14:136–43. 10.1002/cyto.990140205 7679962

[22] Reipert S , Gruber D, Cyran N et al. Freeze substitution accelerated via agitation: new prospects for ultrastructural studies of lichen symbionts and their extracellular matrix. *Plan Theory* 2023;12:4039. 10.3390/plants12234039 PMC10708280 38068675

[23] Hartman LM , van Oppen MJH, Blackall LL. Microbiota characterization of Exaiptasia diaphana from the great barrier reef. *Anim Microbiome* 2020;2:10. 10.1186/s42523-020-00029-5 33499977 PMC7807684

[24] Hume BCC , Ziegler M, Poulain J et al. An improved primer set and amplification protocol with increased specificity and sensitivity targeting the Symbiodinium ITS2 region. *PeerJ* 2018;6:e4816. 10.7717/peerj.4816 29844969 PMC5970565

[25] Hugerth LW , Muller EEL, Hu YOO et al. Systematic design of 18S rRNA gene primers for determining eukaryotic diversity in microbial consortia. *PLoS One* 2014;9:e95567. 10.1371/journal.pone.0095567 24755918 PMC3995771

[26] Hadziavdic K , Lekang K, Lanzen A et al. Characterization of the 18S rRNA gene for designing universal eukaryote specific primers. *PLoS One* 2014;9:e87624. 10.1371/journal.pone.0087624 24516555 PMC3917833

[27] Maire J , Blackall LL, van Oppen MJH. Microbiome characterization of defensive tissues in the model anemone Exaiptasia diaphana. *BMC Microbiol* 2021;21:152. 10.1186/s12866-021-02211-4 34020587 PMC8140459

[28] Hume BCC , Smith EG, Ziegler M et al. SymPortal: a novel analytical framework and platform for coral algal symbiont next-generation sequencing ITS2 profiling. *Mol Ecol Resour* 2019;19:1063–80. 10.1111/1755-0998.13004 30740899 PMC6618109

[29] Bolyen E , Rideout JR, Dillon MR et al. Reproducible, interactive, scalable and extensible microbiome data science using QIIME 2. *Nat Biotechnol* 2019;37:852–7. 10.1038/s41587-019-0209-9 31341288 PMC7015180

[30] Martin M . Cutadapt removes adapter sequences from high-throughput sequencing reads. *EMBnetjournal* 2011;17:10. 10.14806/ej.17.1.200

[31] Callahan BJ , McMurdie PJ, Rosen MJ et al. DADA2: high-resolution sample inference from Illumina amplicon data. *Nat Methods* 2016;13:581–3. 10.1038/nmeth.3869 27214047 PMC4927377

[32] Guillou L , Bachar D, Audic S et al. The Protist ribosomal reference database (PR2): a catalog of unicellular eukaryote small sub-unit rRNA sequences with curated taxonomy. *Nucleic Acids Res* 2013;41:D597–604. 10.1093/nar/gks1160 23193267 PMC3531120

[33] Bokulich NA , Kaehler BD, Rideout JR et al. Optimizing taxonomic classification of marker-gene amplicon sequences with QIIME 2’s q2-feature-classifier plugin. *Microbiome* 2018;6:90. 10.1186/s40168-018-0470-z 29773078 PMC5956843

[34] McMurdie PJ , Holmes S. Phyloseq: an R package for reproducible interactive analysis and graphics of microbiome census data. Watson M (ed.). PLoS One 2013;8:e61217, 10.1371/journal.pone.0061217. 23630581 PMC3632530

[35] De Coster W , Rademakers R. NanoPack2: population-scale evaluation of long-read sequencing data. *Bioinformatics* 2023;39:btad311. 10.1093/bioinformatics/btad311 37171891 PMC10196664

[36] Kolmogorov M , Bickhart DM, Behsaz B et al. metaFlye: scalable long-read metagenome assembly using repeat graphs. *Nat Methods* 2020;17:1103–10. 10.1038/s41592-020-00971-x 33020656 PMC10699202

[37] Von Meijenfeldt FAB , Arkhipova K, Cambuy DD et al. Robust taxonomic classification of uncharted microbial sequences and bins with CAT and BAT. *Genome Biol* 2019;20:217. 10.1186/s13059-019-1817-x 31640809 PMC6805573

[38] Chaumeil PA , Mussig AJ, Hugenholtz P et al. GTDB-Tk: a toolkit to classify genomes with the genome taxonomy database. *Bioinformatics* 2020;36:1925–7. 10.1093/bioinformatics/btz848 PMC7703759 31730192

[39] Lamurias A , Sereika M, Albertsen M et al. Metagenomic binning with assembly graph embeddings. *Bioinformatics* 2022;38:4481–7. 10.1093/bioinformatics/btac557 35972375 PMC9525014

[40] Bolger AM , Lohse M, Usadel B. Trimmomatic: a flexible trimmer for Illumina sequence data. *Bioinforma Oxf Engl* 2014;30:2114–20. 10.1093/bioinformatics/btu170 PMC4103590 24695404

[41] Langmead B , Salzberg SL. Fast gapped-read alignment with bowtie 2. *Nat Methods* 2012;9:357–9. 10.1038/nmeth.1923 22388286 PMC3322381

[42] Li H , Handsaker B, Wysoker A et al. The sequence alignment/map format and SAMtools. *Bioinformatics* 2009;25:2078–9. 10.1093/bioinformatics/btp352 19505943 PMC2723002

[43] Walker BJ , Abeel T, Shea T et al. Pilon: an integrated tool for comprehensive microbial variant detection and genome assembly improvement. *PLoS One* 2014;9:e112963. 10.1371/journal.pone.0112963 25409509 PMC4237348

[44] Köstlbacher S , Collingro A, Halter T et al. Coevolving plasmids drive gene flow and genome plasticity in host-associated intracellular bacteria. *Curr Biol* 2021;31:346–357.e3. 10.1016/j.cub.2020.10.030 33157023 PMC7846284

[45] Li H . Minimap2: pairwise alignment for nucleotide sequences. *Bioinformatics* 2018;34:3094–100. 10.1093/bioinformatics/bty191 29750242 PMC6137996

[46] Wick RR , Judd LM, Gorrie CL et al. Unicycler: resolving bacterial genome assemblies from short and long sequencing reads. *PLoS Comput Biol* 2017;13:e1005595. 10.1371/journal.pcbi.1005595 28594827 PMC5481147

[47] Chklovski A , Parks DH, Woodcroft BJ et al. CheckM2: a rapid, scalable and accurate tool for assessing microbial genome quality using machine learning. *Nat Methods* 2023;20:1203–12. 10.1038/s41592-023-01940-w 37500759

[48] Dharamshi JE , Gaarslev N, Steffen K et al. Genomic diversity and biosynthetic capabilities of sponge-associated chlamydiae. *ISME J* 2022;16:2725–40. 10.1038/s41396-022-01305-9 36042324 PMC9666466

[49] Katoh K , Standley DM. MAFFT multiple sequence alignment software version 7: improvements in performance and usability. *Mol Biol Evol* 2013;30:772–80. 10.1093/molbev/mst010 23329690 PMC3603318

[50] Criscuolo A , Gribaldo S. BMGE (block mapping and gathering with entropy): a new software for selection of phylogenetic informative regions from multiple sequence alignments. *BMC Evol Biol* 2010;10:210. 10.1186/1471-2148-10-210 20626897 PMC3017758

[51] Minh BQ , Schmidt HA, Chernomor O et al. IQ-TREE 2: new models and efficient methods for phylogenetic inference in the genomic era. *Mol Biol Evol* 2020;37:1530–4. 10.1093/molbev/msaa015 32011700 PMC7182206

[52] Kalyaanamoorthy S , Minh BQ, Wong TKF et al. ModelFinder: fast model selection for accurate phylogenetic estimates. *Nat Methods* 2017;14:587–9. 10.1038/nmeth.4285 28481363 PMC5453245

[53] Hoang DT , Chernomor O, von Haeseler A et al. UFBoot2: improving the ultrafast bootstrap approximation. *Mol Biol Evol* 2018;35:518–22. 10.1093/molbev/msx281 29077904 PMC5850222

[54] Guindon S , Dufayard J-F, Lefort V et al. New algorithms and methods to estimate maximum-likelihood phylogenies: assessing the performance of PhyML 3.0. *Syst Biol* 2010;59:307–21. 10.1093/sysbio/syq010 20525638

[55] Wang H-C , Minh BQ, Susko E et al. Modeling site heterogeneity with posterior mean site frequency profiles accelerates accurate Phylogenomic estimation. *Syst Biol* 2018;67:216–35. 10.1093/sysbio/syx068 28950365

[56] Letunic I , Bork P. Interactive tree of life (iTOL) v5: an online tool for phylogenetic tree display and annotation. *Nucleic Acids Res* 2021;49:W293–6. 10.1093/nar/gkab301 33885785 PMC8265157

[57] Schwengers O , Jelonek L, Dieckmann MA et al. Bakta: rapid and standardized annotation of bacterial genomes via alignment-free sequence identification. *Microb Genomics* 2021;7:000685. 10.1099/mgen.0.000685 PMC8743544 34739369

[58] Kanehisa M , Sato Y. KEGG mapper for inferring cellular functions from protein sequences. *Protein Sci Publ Protein Soc* 2020;29:28–35. 10.1002/pro.3711 PMC6933857 31423653

[59] Cantalapiedra CP , Hernández-Plaza A, Letunic I et al. eggNOG-mapper v2: functional annotation, Orthology assignments, and domain prediction at the metagenomic scale. *Mol Biol Evol* 2021;38:5825–9. 10.1093/molbev/msab293 34597405 PMC8662613

[60] Jones P , Binns D, Chang HY et al. InterProScan 5: genome-scale protein function classification. *Bioinformatics* 2014;30:1236–40. 10.1093/bioinformatics/btu031 24451626 PMC3998142

[61] Blin K , Shaw S, Augustijn HE et al. antiSMASH 7.0: new and improved predictions for detection, regulation, chemical structures and visualisation. *Nucleic Acids Res* 2023;51:W46–50. 10.1093/nar/gkad344 37140036 PMC10320115

[62] Altschul SF , Madden TL, Schäffer AA et al. Gapped BLAST and PSI-BLAST: a new generation of protein database search programs. *Nucleic Acids Res* 1997;25:3389–402. 10.1093/nar/25.17.3389 9254694 PMC146917

[63] Collingro A , Tischler P, Weinmaier T et al. Unity in variety—the pan-genome of the Chlamydiae. *Mol Biol Evol* 2011;28:3253–70. 10.1093/molbev/msr161 21690563 PMC3247790

[64] Stephens RS , Kalman S, Lammel C et al. Genome sequence of an obligate intracellular pathogen of humans: chlamydia trachomatis. *Science* 1998;282:754–9. 10.1126/science.282.5389.754 9784136

[65] Major P , Embley TM, Williams TA. Phylogenetic diversity of NTT nucleotide transport proteins in free-living and parasitic bacteria and eukaryotes. *Genome Biol Evol* 2017;9:480–7. 10.1093/gbe/evx015 28164241 PMC5381601

[66] Emms DM , Kelly S. OrthoFinder: phylogenetic orthology inference for comparative genomics. *Genome Biol* 2019;20:238. 10.1186/s13059-019-1832-y 31727128 PMC6857279

[67] Jumper J , Evans R, Pritzel A et al. Highly accurate protein structure prediction with AlphaFold. *Nature* 2021;596:583–9. 10.1038/s41586-021-03819-2 34265844 PMC8371605

[68] Meng EC , Goddard TD, Pettersen EF et al. UCSF ChimeraX: tools for structure building and analysis. *Protein Sci Publ Protein Soc* 2023;32:e4792. 10.1002/pro.4792 PMC10588335 37774136

[69] Burley SK , Bhikadiya C, Bi C et al. RCSB protein data Bank (RCSB.org): delivery of experimentally-determined PDB structures alongside one million computed structure models of proteins from artificial intelligence/machine learning. *Nucleic Acids Res* 2023;51:D488–508. 10.1093/nar/gkac1077 36420884 PMC9825554

[70] Elwell C , Mirrashidi K, Engel J. Chlamydia cell biology and pathogenesis. *Nat Rev Microbiol* 2016;14:385–400. 10.1038/nrmicro.2016.30 27108705 PMC4886739

[71] Heinz E , Rockey DD, Montanaro J et al. Inclusion membrane proteins of Protochlamydia amoebophila UWE25 reveal a conserved mechanism for host cell interaction among the Chlamydiae. *J Bacteriol* 2010;192:5093–102. 10.1128/JB.00605-10 20675479 PMC2944539

[72] Benamar S , Bou Khalil JY, Blanc-Tailleur C et al. Developmental cycle and genome analysis of Protochlamydia massiliensis sp. nov. a new species in the Parachlamydiacae family. *Front Cell Infect Microbiol* 2017;7:385. 10.3389/fcimb.2017.00385 28913180 PMC5583166

[73] Hedlund BP , Chuvochina M, Hugenholtz P et al. SeqCode: a nomenclatural code for prokaryotes described from sequence data. *Nat Microbiol* 2022;7:1702–8. 10.1038/s41564-022-01214-9 36123442 PMC9519449

[74] Dudiak BM , Nguyen TM, Needham D et al. Inhibition of the futalosine pathway for menaquinone biosynthesis suppresses chlamydia trachomatis infection. *FEBS Lett* 2021;595:2995–3005. 10.1002/1873-3468.14223 34741525 PMC9980418

[75] Krumbholz J , Ishida K, Baunach M et al. Deciphering chemical mediators regulating specialized metabolism in a symbiotic cyanobacterium. *Angew Chem Int Ed Engl* 2022;61:e202204545. 10.1002/anie.202204545 35403785 PMC9324945

[76] Köstlbacher S , Collingro A, Halter T et al. Pangenomics reveals alternative environmental lifestyles among chlamydiae. *Nat Commun* 2021;12:4021. 10.1038/s41467-021-24294-3 34188040 PMC8242063

[77] Haferkamp I , Schmitz-Esser S, Wagner M et al. Tapping the nucleotide pool of the host: novel nucleotide carrier proteins of Protochlamydia amoebophila. *Mol Microbiol* 2006;60:1534–45. 10.1111/j.1365-2958.2006.05193.x 16796686 PMC1513512

[78] Knab S , Mushak TM, Schmitz-Esser S et al. Nucleotide parasitism by Simkania negevensis (Chlamydiae). *J Bacteriol* 2011;193:225–35. 10.1128/JB.00919-10 20971898 PMC3019940

[79] Greub G , Raoult D. History of the ADP/ATP-translocase-encoding gene, a parasitism gene transferred from a Chlamydiales ancestor to plants 1 billion years ago. *Appl Environ Microbiol* 2003;69:5530–5. 10.1128/AEM.69.9.5530-5535.2003 12957942 PMC194985

[80] Rucks EA . Type III secretion in chlamydia. *Microbiol Mol Biol Rev* 2023;87:e00034–23. 37358451 10.1128/mmbr.00034-23 PMC10521360

[81] Greub G , Collyn F, Guy L et al. A genomic island present along the bacterial chromosome of the Parachlamydiaceae UWE25, an obligate amoebal endosymbiont, encodes a potentially functional F-like conjugative DNA transfer system. *BMC Microbiol* 2004;4:48. 10.1186/1471-2180-4-48 15615594 PMC548262

[82] Smith L , Hillman J. Therapeutic potential of type a (I) lantibiotics, a group of cationic peptide antibiotics. *Curr Opin Microbiol* 2008;11:401–8. 10.1016/j.mib.2008.09.008 18848642 PMC2603291

[83] Arthofer P , Delafont V, Willemsen A et al. Defensive symbiosis against giant viruses in amoebae. *Proc Natl Acad Sci* 2022;119:e2205856119. 10.1073/pnas.2205856119 36037367 PMC9457554

[84] Gupta TK , Klumpe S, Gries K et al. Structural basis for VIPP1 oligomerization and maintenance of thylakoid membrane integrity. *Cell* 2021;184:3643–3659.e23. 10.1016/j.cell.2021.05.011 34166613

[85] Zhang L , Kato Y, Otters S et al. Essential role of VIPP1 in chloroplast envelope maintenance in Arabidopsis. *Plant Cell* 2012;24:3695–707. 10.1105/tpc.112.103606 23001039 PMC3480296

[86] Zhang L , Kondo H, Kamikubo H et al. VIPP1 has a disordered C-terminal tail necessary for protecting photosynthetic membranes against stress. *Plant Physiol* 2016;171:1983–95. 10.1104/pp.16.00532 27208228 PMC4936581

[87] Maire J , Tandon K, Collingro A et al. Colocalization and potential interactions of Endozoicomonas and chlamydiae in microbial aggregates of the coral Pocillopora acuta. *Sci Adv* 2023;9:eadg0773. 10.1126/sciadv.adg0773 37196086 PMC11809670

[88] Damjanovic K , Blackall LL, Peplow LM et al. Assessment of bacterial community composition within and among Acropora loripes colonies in the wild and in captivity. *Coral Reefs* 2020;39:1245–55. 10.1007/s00338-020-01958-y

[89] Maire J , Collingro A, Horn M et al. Chlamydiae in corals: shared functional potential despite broad taxonomic diversity. *ISME Commun* 2024;4:ycae054. 10.1093/ismeco/ycae054 38707840 PMC11070183

[90] McCauley M , Goulet TL, Jackson CR et al. Systematic review of cnidarian microbiomes reveals insights into the structure, specificity, and fidelity of marine associations. *Nat Commun* 2023;14:4899. 10.1038/s41467-023-39876-6 37580316 PMC10425419

[91] Dharamshi JE , Tamarit D, Eme L et al. Marine sediments illuminate Chlamydiae diversity and evolution. *Curr Biol* 2020;30:1032–1048.e7. 10.1016/j.cub.2020.02.016 32142706

[92] Davy SK , Allemand D, Weis VM. Cell biology of cnidarian-dinoflagellate symbiosis. *Microbiol Mol Biol Rev MMBR* 2012;76:229–61. 10.1128/MMBR.05014-11 22688813 PMC3372257

[93] Lee STM , Davy SK, Tang S-L et al. Successive shifts in the microbial community of the surface mucus layer and tissues of the coral Acropora muricata under thermal stress. *FEMS Microbiol Ecol* 2015;91:fiv142. 10.1093/femsec/fiv142 26564958

[94] Fong J , Deignan LK, Bauman AG et al. Contact- and water-mediated effects of macroalgae on the physiology and microbiome of three indo-Pacific coral species. *Front Mar Sci* 2020;6:831. 10.3389/fmars.2019.00831

[95] Pollock FJ , McMinds R, Smith S et al. Coral-associated bacteria demonstrate phylosymbiosis and cophylogeny. *Nat Commun* 2018;9:4921. 10.1038/s41467-018-07275-x 30467310 PMC6250698

[96] Doering T , Wall M, Putchim L et al. Towards enhancing coral heat tolerance: a “microbiome transplantation” treatment using inoculations of homogenized coral tissues. *Microbiome* 2021;9:102. 10.1186/s40168-021-01053-6 33957989 PMC8103578

[97] Zhang Y , Yang Q, Zhang Y et al. Shifts in abundance and network complexity of coral bacteria in response to elevated ammonium stress. *Sci Total Environ* 2021;768:144631. 10.1016/j.scitotenv.2020.144631 33434804

[98] Jeong HJ , Yoo YD, Kang NS et al. Heterotrophic feeding as a newly identified survival strategy of the dinoflagellate Symbiodinium. *Proc Natl Acad Sci USA* 2012;109:12604–9. 10.1073/pnas.1204302109 22814379 PMC3412036

[99] Wong WF , Chambers JP, Gupta R et al. Chlamydia and its many ways of escaping the host immune system. *J Pathog* 2019;2019:1–9. 10.1155/2019/8604958 PMC6699355 31467721

[100] Massé A , Detang J, Duval C et al. Bacterial microbiota of Ostreobium, the coral-isolated chlorophyte Ectosymbiont, at contrasted salinities. *Microorganisms* 2023;11:1318. 10.3390/microorganisms11051318 37317290 PMC10223477

[101] Pushpakumara BLDU , Tandon K, Willis A et al. The bacterial microbiome of the coral skeleton algal symbiont Ostreobium shows preferential associations and signatures of Phylosymbiosis. *Microb Ecol* 2023;86:2032–46. 10.1007/s00248-023-02209-7 37002423 PMC10497448

[102] Davison HR , Hurst GDD. Hidden from plain sight: novel Simkaniaceae and Rhabdochlamydiaceae diversity emerging from screening genomic and metagenomic data. *Syst Appl Microbiol* 2023;46:126468. 10.1016/j.syapm.2023.126468 37847957

