# Supplementary material for: Chlamydiae as symbionts of photosynthetic dinoflagellates
Source: ISME J. 2024 Jul 24;18(1):wrae139. doi: 10.1093/ismejo/wrae139 (PMC11317633; doi:10.1093/ismejo/wrae139)
Supplement: supplementary_algichlamydia_revision3_wrae139 [file supplementary_algichlamydia_revision3_wrae139.docx]

**Supplementary text**

**Taxonomy of *Cladocopium* sp. SCF049.01**

The *Cladocopium* genus has recently seen multiple taxonomic updates, including the formal description of four new species since 2021 [1,2]. Therefore, we assessed whether the culture used in the present study, SCF049.01, fell within any of these newly described species. Phylogeny based on five genetic markers, ITS2, LSU, cp23S, *cox1*, and *cob* placed SCF049.01 within the C1 radiation; it is most closely related to the newly described *C. proliferum* and *C. vulgare* (Figure S1A). Metabarcoding of the ITS2 region confirmed its ITS2 type as C1 (Figure S1B). Surprisingly, it is more distantly related to *C. latusorum* and *C. pacificum*, both isolated from *Pocillopora* corals like SCF049.01. Nonetheless, its phylogenetic placement is not close enough to *C. proliferum* or *C. vulgare* for it to be placed in either species, suggesting that SCF049.01 belongs to an undescribed species of the C1 radiation within the *Cladocopium* genus.

**Supplementary material and methods**

**FISH on *Symbiodiniaceae* cells**

*Symbiodiniaceae* cells were fixed in ice-cold 66% ethanol and photobleached as previously described [3]. Teflon-printed microscope slides (ProSciTech) were coated with poly-L-lysine solution (0.01%) in PBS by aliquoting the solution onto printed wells, incubated at 37°C for 3 h, then thrice washed in sterile Milli-Q water and air dried. Sample aliquots of 5-10 μL were pipetted into a well on treated ten-well slide and FISH was then performed as previously described [3]. Hybridization was in 16 μL buffer (0.9M NaCl, 20 mM Tris-HCL pH 7.2, 25% formamide, 0.01% SDS) and 2 μL of the 16S rRNA-targeting, chlamydiae-specific probe Chls523 (CCTCCGTATTACCGCAGC; [4]), conjugated with a DOPE-Cy3 fluorophore, along with 2 µL of a competitor probe (CCTCCGTATTACCGCGGC; [4]), both at a final concentration of 5 ng/μL. The antisense nonEUB probe (ACATCCTACGGGAGG) [5] was also used as a negative control in the same conditions. NaCl concentration in washing buffer was 0.149 M. Slides were mounted with CitiFluor™ CFM3 mounting medium (Hatfield, PA, USA) and stored in the dark at -20 °C until observation by CLSM.

**Confocal Laser Scanning Microscopy (CLSM)**

Observations were made on a Nikon AIR CLSM (Nikon, Tokyo, Japan) with the NIS325 Element software. Virtual band mode was used to acquire variable emission bandwidth to tailor acquisition for specific fluorophores. An oil immersion objective of 60X magnification and 1.4 numerical aperture was used for image acquisition. The fluorophore Cy3 was excited using the 561 nm laser line, and the *Symbiodiniaceae* autofluorescence using the 488 nm laser line, with a detection range of 570-635 nm for Cy3, and 670-720 nm for *Symbiodiniaceae*. Z-stacks were acquired using Z steps of 0.2 μM. Nd2 files were processed using ImageJ. Single photos were extracted from Z-stacks. Each photo was selected in the center of the Z-stack and covers 0.2 µm. As *Symbiodiniaceae* cells have a diameter of around 8-10 µm, FISH signal present within a *Symbiodiniaceae* cell represents intracellular bacteria.

**Transmission electron microscopy (TEM)**

Fourteen days following media change, as described above, *Cladocopium* sp. SCF049.01 cells were harvested by centrifugation at 5000 × g for 5 minutes, washed once in 1× PBS, and resuspended in 1× PBS. Preparation of samples for TEM was performed as previously described [6] with some adaptations mentioned below. *Cladocopium* sp. suspensions were transferred into type A carriers (LEICA Microsystems, Vienna, Austria) (3 mm in diameter; 100 mm in depth) and covered with the flat surface of a type B carrier. Notably, the carriers were coated with 1-hexadecene (Merck Sharp & Dome Corp., Kenilworth, NJ, USA) prior to use, and the inside volume was filled up with 10% bovine serum albumin (BSA) (PAA Laboratories GmbH, Cölbe, Germany). The carrier sandwich was inserted into the middle plate of a sample cartridge and frozen with a high-pressure freezer HPM100 (LEICA Microsystems, Austria).

Freeze substitution was performed in an automatic AFS2 system (Leica Microsystems, Vienna, Austria), equipped with an agitation module (Helmuth Goldammer, Cryomodultech e.U., Vienna, Austria) as previously described [6]. Carriers containing the high pressure-frozen samples were placed onto frozen substitution medium, consisting of 1% OsO_4_ in anhydrous acetone in Sarstedt tubes. Without interrupting the cool chain, the Sarstedt tubes were inserted in the tube holders of the agitation module located in the precooled chamber of the AFS2. The rotor for sample agitation was set at medium speed at 15 V. Freeze substitution was carried out under agitation at −85°C.

The infiltration with low-viscosity epoxy resin (Agar Scientific, Stansted, UK) was performed stepwise in solvent/resin mixtures, as follows: 1 part resin in 3 parts acetone and 1 part resin in 1 part acetone for 1 h each, followed by 3 parts resin in 1 part acetone for 1.5 h. After two consecutive infiltration steps with pure resin for 1 h each, the samples were polymerized at 65°C for 36 h. Sectioning and observation was performed as previously described [6].

**FISH and flow cytometry**

FISH and flow cytometry analyses were conducted as previously described [3]. Each sample was separated into two aliquots. FISH was performed on both aliquots directly in the tube, one receiving no probe during the hybridization step, while the other received the 16S rRNA-targeting, chlamydiae-specific probe Chls523 (CCTCCGTATTACCGCAGC; [4]), conjugated with a DOPE-Cy3 fluorophore, along with a competitor probe (CCTCCGTATTACCGCGGC; [4]), both at a final concentration of 5 ng/μL. Samples were analyzed on a  CytoFLEX LX flow cytometer (Beckman Coulter Inc, USA). For each sample, the unstained aliquot was used to determine cell autofluorescence in the FISH-specific channel (561 nm laser; emission 610 ± 10 nm), and a quadrant was drawn to encompass unstained cells on one side (autofluorescence only), and stained cells on the other side (autofluorescence + FISH signal) [3]. The proportion of cells above the line was interpreted as the proportion of cells stained by FISH.

**Determination of chlamydial copy numbers by digital PCR**

To determine chlamydial cell numbers present outside (*i.e.,* in the supernatant) of the host and intracellular in *Cladocopium* sp., we performed digital PCR (dPCR). Six separate culture flasks containing 9 mL of cultures were harvested by shaking and subsequently centrifuged at 5,000 × *g* for 5 min. Each sample was then separated in supernatant (n = 6) and cellular fraction (n = 6). The supernatant was filtered through 5 µm and 1.2 µm filters to retrieve extracellular chlamydiae only. *Cladocopium* sp. cells in the cellular fraction were washed once in IMK medium and subsequently counted with a LUNA-FX7 cell counter (Logos Biosystems). DNA was extracted from all samples using the DNeasy PowerSoil Pro Kit (Qiagen) according to the manufacturer’s instructions. Only the initial cell lysis step was carried out in a FastPrep-24 instrument in lysing matrix A tubes (both MP Biomedicals) under different settings for the supernatant and cellular fraction samples, respectively (supernatant: 4.0 m/sec, 30 seconds; cellular fraction: 5.0 m/sec, 40 seconds). Resulting DNA concentrations were determined with Nanodrop and diluted to roughly use 0.5 ng/µL DNA as template for the dPCR. Digital PCR was performed using the QIAcuity EvaGreen PCR kit (Qiagen) and the chlamydiae-specific primer pair Chl40F (5’ CRG CGT GGA TGA GGC AT 3’) and Chl523R (5’ CCY YMC GTA TTA CCG CAG CT 3’) targeting the 16S rRNA gene on a QIAcuity One digital PCR device (Qiagen) as recommended by the manufacturer. The cycling conditions were 95°C for 2 min; 35 cycles of 95°C for 30 sec, 64.5°C for 45 sec, 72°C for 1 min; 40°C for 5 min with imaging at an exposure time of 350 ms and a gain of 3. Direct quantification data were acquired with the QIAcuity Software Suite 2.2.0.26 (Qiagen) and chlamydial 16S rRNA copy numbers per mL were calculated based on these data. As the *A. australiensis* Cla049 genome contains only one copy of the 16S rRNA gene, the determined gene copy numbers per mL correspond to chlamydial cell numbers per mL. In the cellular fraction, the chlamydial cell numbers per mL were normalized to the *Cladocopium* sp. cell counts.

***Symbiodiniaceae* taxonomy**

To assess the taxonomical placement of SCF049.01, DNA was extracted from ~1 × 10^6^ cells as described in the section ‘DNA extractions for metabarcoding’. Four conserved DNA markers (internal transcribed space region 2 [ITS2], large ribosomal subunit [LSU], partial chloroplast cp23S, mitochondrial cytochrome b [*cob*], and mitochondrial cytochrome oxidase 1 [*cox1*]) were PCR-amplified, sequenced, and concatenated as previously described [1]. The sequences were trimmed and aligned (MAFFT alignment) with other *Cladocopium* spp. concatenated sequences obtained from previous studies [1,7] using Geneious Prime v2019.1.3. The full alignment was stripped of columns containing 99% or more gaps. This alignment was used to generate a maximum likelihood phylogenetic tree with 1000 ultrafast bootstraps using IQ-TREE v2.2.2.3 [8] with the best model HKY+F+G4, selected by ModelFinder wrapped in IQ-TREE [9].


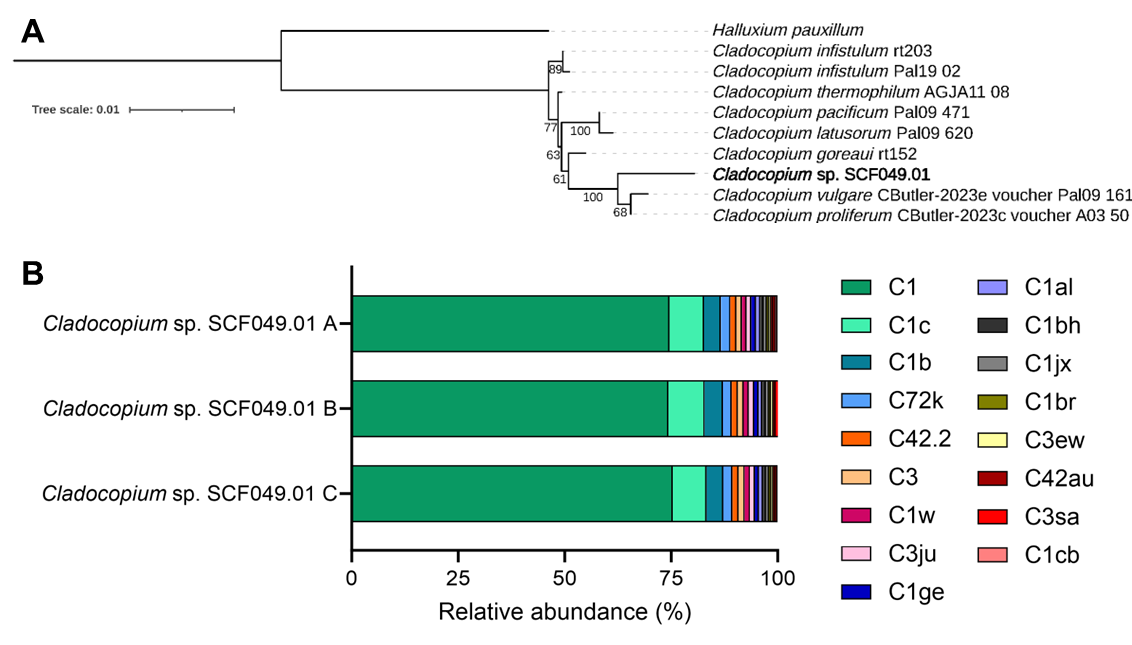


**Figure S1:** Taxonomic placement of *Cladocopium* sp. SCF049.01. **A:** Maximum likelihood phylogeny from aligned concatenated markers (ITS2, LSU, cp23S, *cob*, and *cox1*), showing the relationship of *Cladocopium* sp. SCF049.01 with other described *Cladocopium* species. Bootstrap values (%) based on 1000 replications are provided. **B:** ITS2 metabarcoding showing *Symbiodiniaceae* ITS2 types of three replicate flasks of *Cladocopium* sp. SCF049.01.


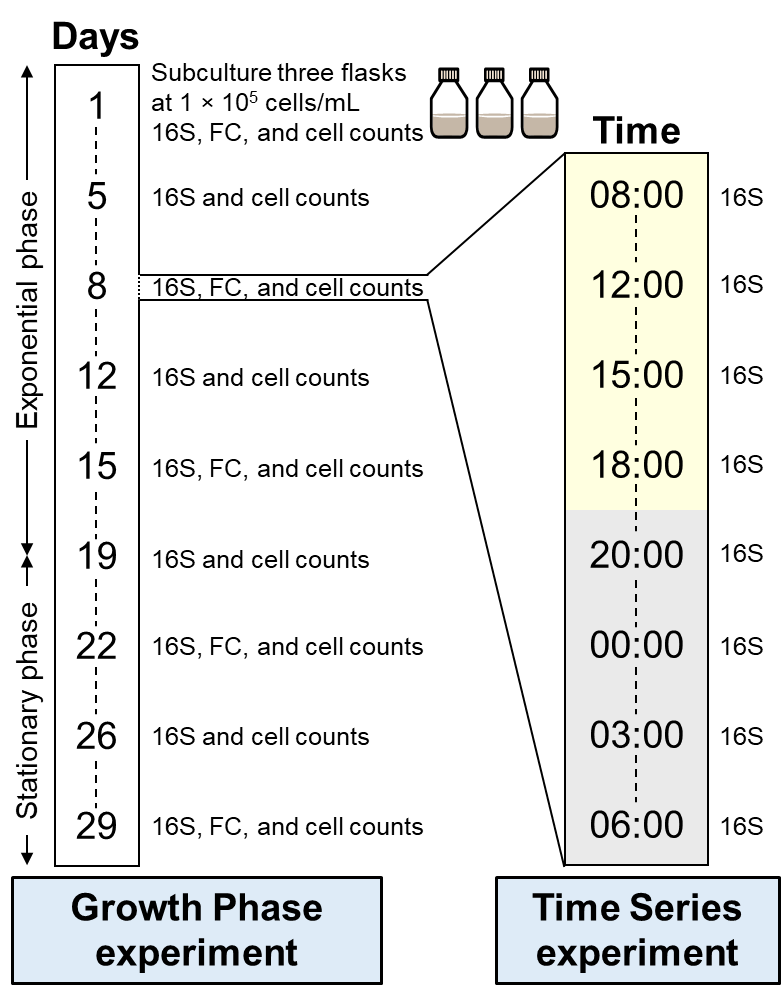


**Figure S2:** Experimental design of the growth phase (left) and time series experiments (right; day 8 of the growth phase experiment). On sampling days or times, flasks were sampled for 16S rRNA gene metabarcoding (‘16S’), flow cytometry (‘FC’, growth phase experiment only) and cell counts (growth phase experiment only). Yellow shading represents daytime sampling and grey shading represents nighttime sampling.


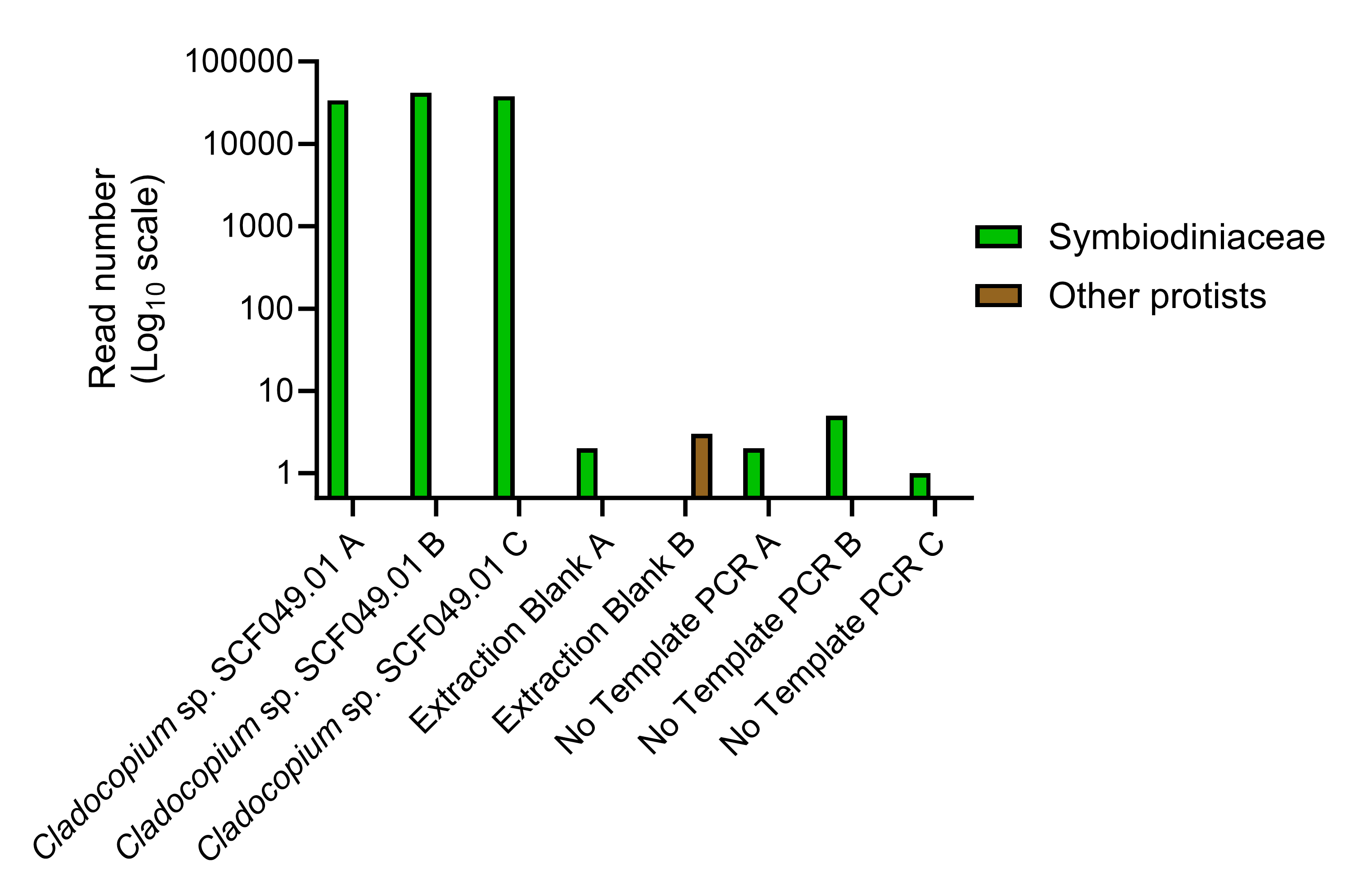


**Figure S3:** 18S rRNA gene metabarcoding showing the protist composition of three replicate flasks of *Cladocopium* sp. SCF049.01. Low levels of cross-contamination during sample preparation may explain the few (< 10) *Symbiodiniaceae* reads in negative controls, although this is negligible compared to the 37,000-42,000 reads obtained from *Symbiodiniaceae* samples.


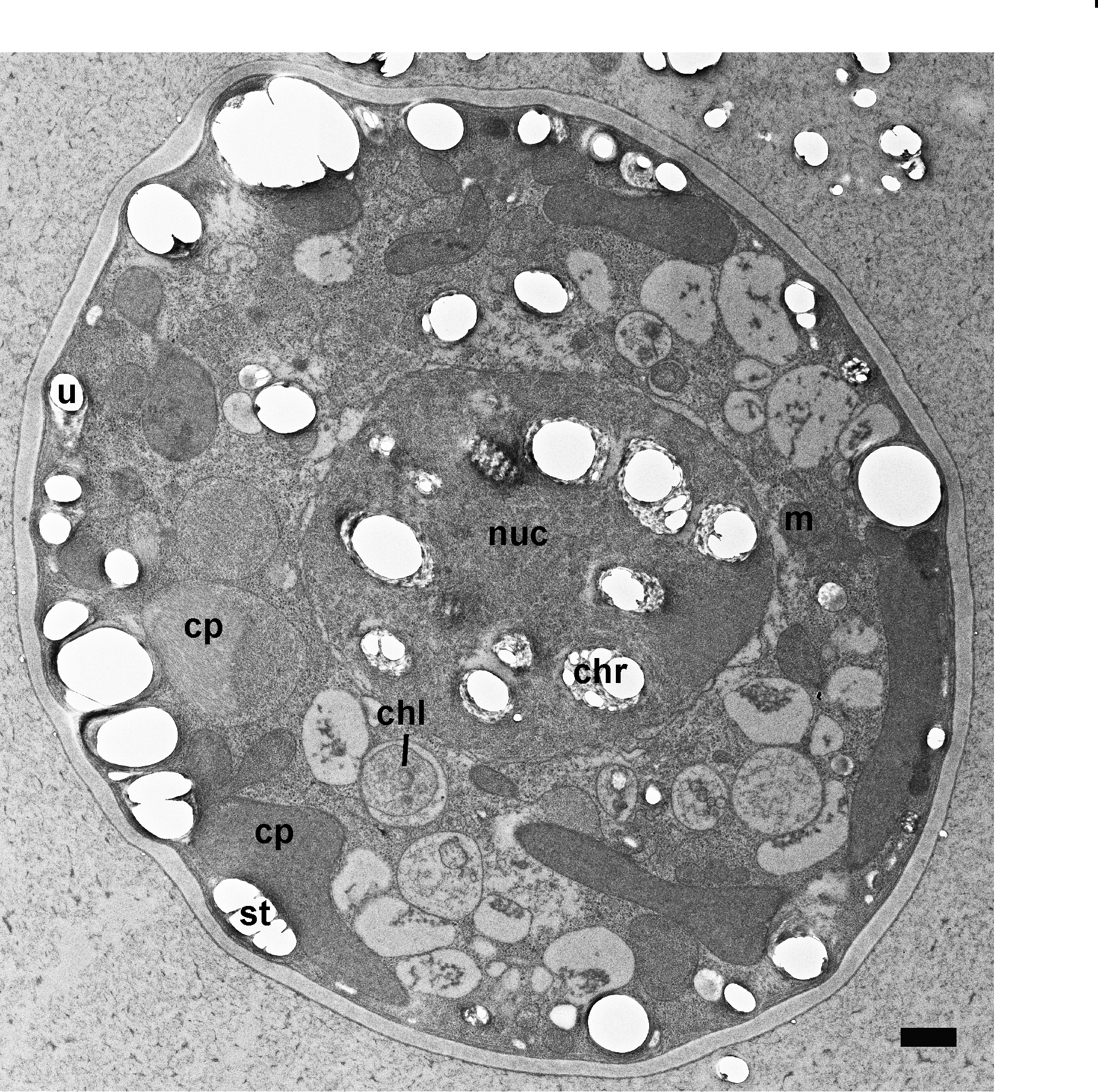


**Figure S4:** Transmission electron micrograph showing a *Cladocopium* sp. cell infected with a chlamydial cell (chl). A detailed image of the chlamydial cell is available in Fig 1E. Abbreviations: chl: chlamydial cell, nuc: nucleus, chr: chromosome, cp: chloroplast, st: starch granule, m: mitochondrium, u: ureic acid crystal. Scale bar: 500 nm.


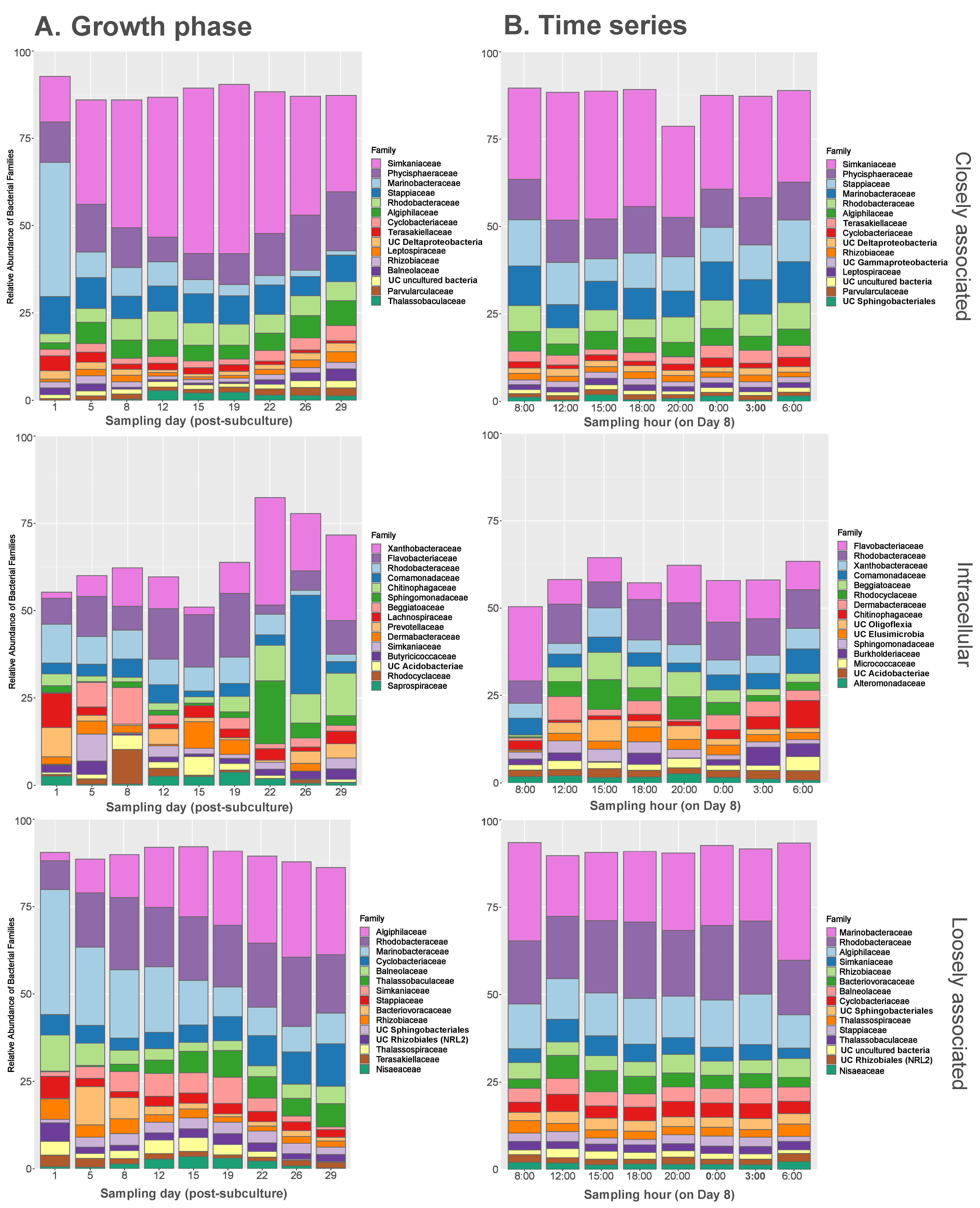


**Figure S5:** Relative abundance of the 15 most abundant bacterial families in *Cladocopium* sp. SCF049.01’s closely associated (top), intracellular (middle), and loosely associated (bottom) communities in the growth phase (A) and time series (B) experiments. For each strain sampling time, three independent replicate flasks were merged. UC: unclassified.

**
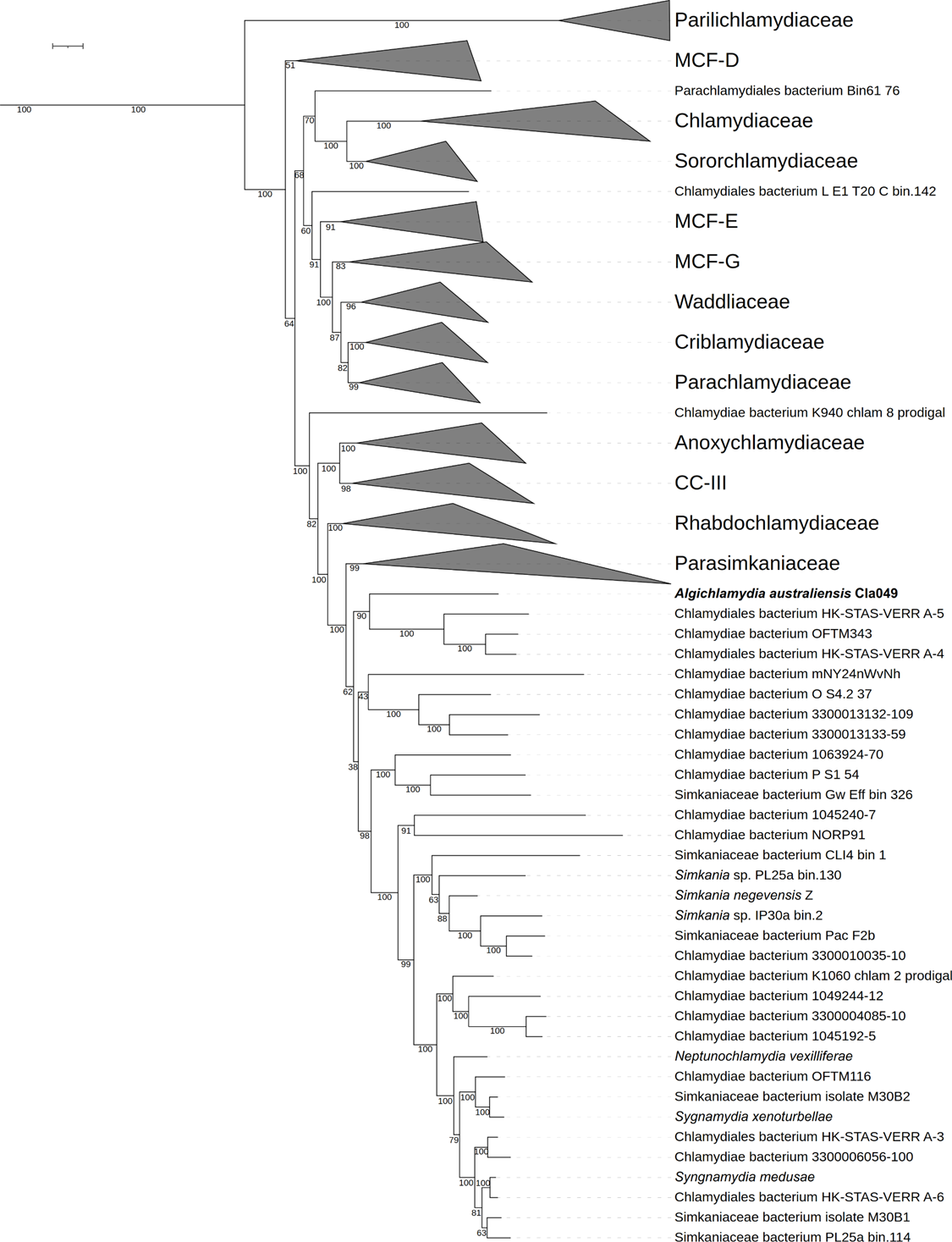
**

**Figure S6:** Chlamydial maximum likelihood phylogeny based on 15 conserved gene markers (Table S4) in 169 chlamydial genomes (Table S3). Confidence values based on 1000 bootstrap replicates are provided. Scale bar represents 0.1 nucleotide substitution per site. This tree was calculated under the posterior mean **s**ite frequency (PMSF) model [10] using the tree in Figure 3 as seed. MCF: metagenomic chlamydial family; CC-III: chlamydiae clade III.

**
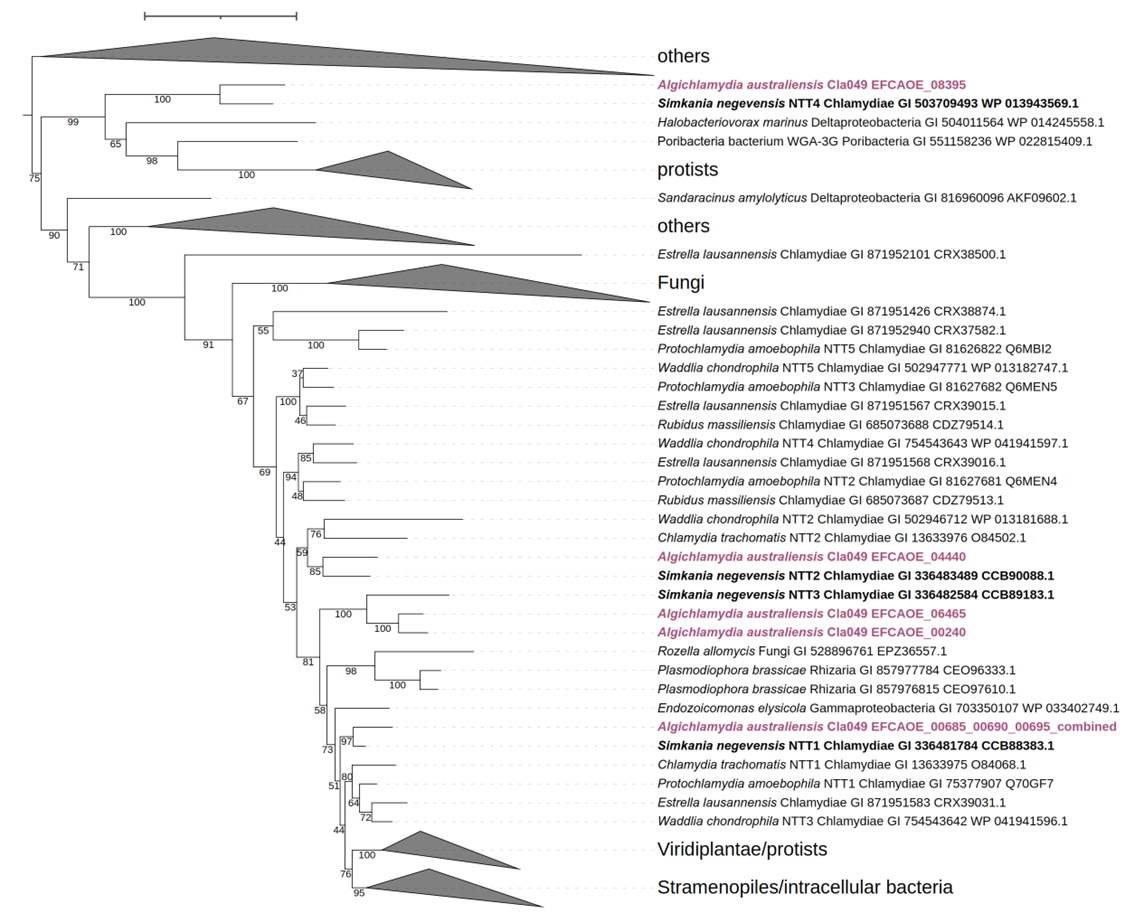
**

**Figure S7:** Phylogenetic tree of nucleotide transport proteins (NTTs) of *Algichlamydia australiensis* Cla049, chlamydiae, and other organisms [11]. Scale bar represents 1 nucleotide substitution per site. Confidence values based on 1000 bootstrap replicates are provided. The NTT1 homolog in *A. australiensis* is a pseudogene.

**
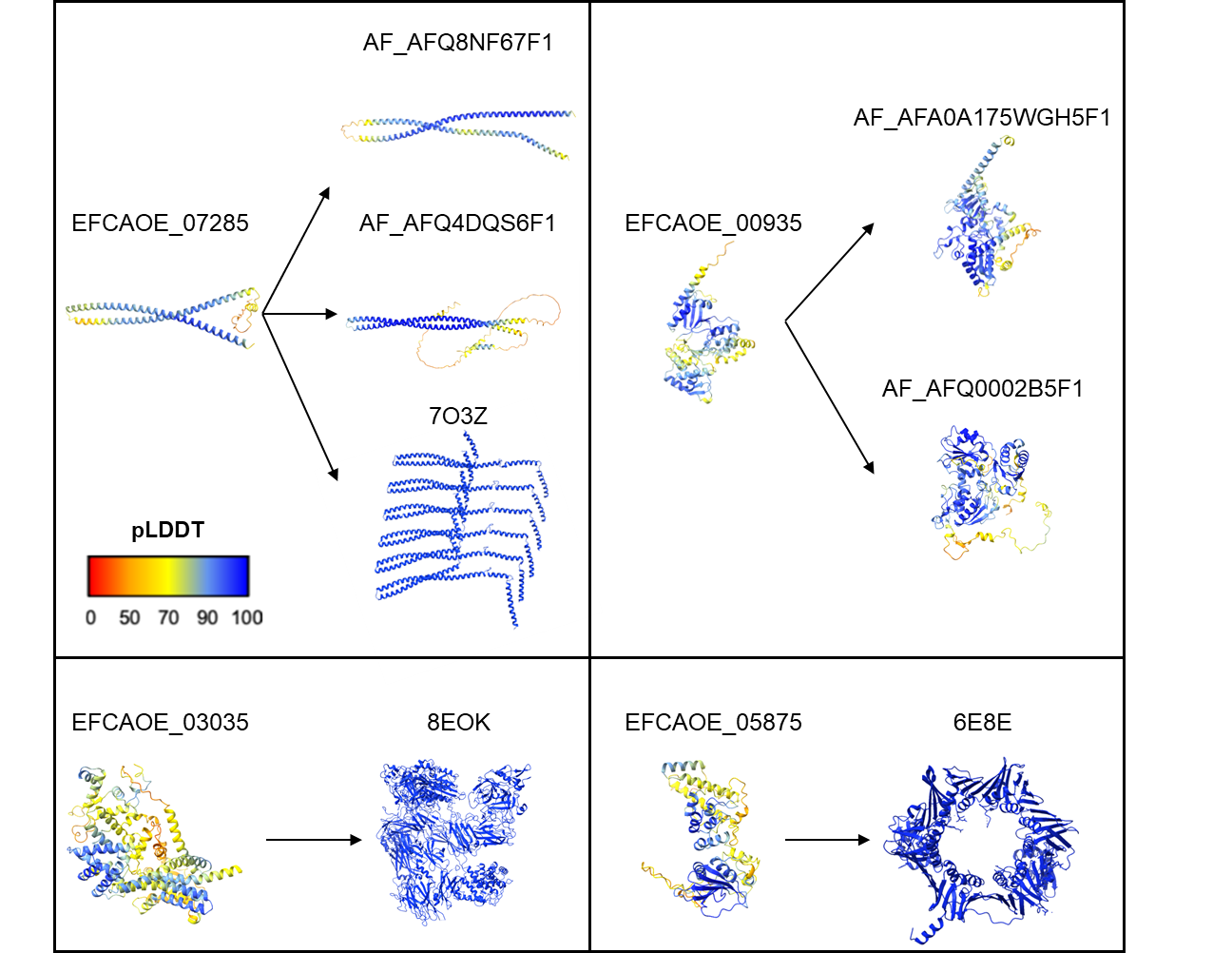
**

**Figure S8:** Predicted structures of four representative genes of OGs unique to *Algichlamydia australiensis* Cla049. Only structures where global pLDDT > 70 are shown here, along with the closest protein structures. See Table S11 for additional information.

**Table S1:** Sequencing statistics for the two 16S rRNA gene metabarcoding experiments analyzed in this study. The first column (“Growth Phase”) refers to Figure 2D and Table S5A, and the second column (“Time Series”) refers to Figure 2E Table S5B.

| **Experiment** | **Growth Phase** | **Time Series** |
| --- | --- | --- |
| **Total Samples (negative controls)** | 98 (17) | 89 (17) |
| **Raw reads** | 3897444 | 3676726 |
| **Reads after merging, denoising and chimera filtering** | 3205712 | 2949638 |
| **Contaminating ASVs** | 29 | 33 |
| **Contamination (%)** | 5.99 | 9.88 |
| **Samples kept for analysis** | 76 | 69 |
| **ASVs after decontamination** | 493 | 2474 |
| **Read per sample** | 40985 | 41642 |

**Table S2:** List of contaminants identified in the 16S rRNA gene metabarcoding data, and their abundance in *Cladocopium* sp. SCF049.01 samples, in the growth phase experiment (A) and the time series experiment (B).

(attached)

**Table S3:** List of chlamydial genomes used for phylogenetic analyses.

(attached)

**Table S4:** Marker Non-supervised Orthologous Group (NOG) proteins used for chlamydial phylogenetic analysis.

| NOG | NOG category | NOG description |
| --- | --- | --- |
| COG0064 | J | Aspartyl-tRNA (Asn)/glutamyl-tRNA (Gln) amidotransferase subunit B |
| COG0092 | J | Ribosomal protein S3 |
| COG0233 | J | Ribosome recycling factor |
| COG0290 | J | Translation initiation factor IF-3 |
| COG0292 | J | Ribosomal protein L20 |
| COG0323 | L | DNA mismatch repair protein MutL |
| COG0335 | J | Ribosomal protein L19 |
| COG0342 | U | Preprotein translocase subunit SecD |
| COG0468 | L | DNA recombination/repair protein RecA |
| COG0532 | J | Translation initiation factor IF-2 |
| COG0536 | DL | GTPase Obg involved in cell cycle, chromosome segregation and ribosome assembly |
| COG0706 | M | Membrane protein insertase YidC |
| COG1185 | J | Polyribonucleotide nucleotidyltransferase Pnp |
| COG1530 | J | Ribonuclease G or E |
| COG1663 | M | Tetraacyldisaccharide-1-P 4’-kinase LpxK |

**Table S5:** Relative abundance of chlamydial ASVs in *Cladocopium* sp. SCF049.01 samples of the growth phase experiment (A) and the time series experiment (B). This data is summarized in Figure 2D-E.

(attached)

**Table S6:** List of conserved genes on chlamydial plasmids and their locus in the genome of *Algichlamydia australiensis* Cla049.

| **Gene** | **Function** | **Locus** | **Contig** |
| --- | --- | --- | --- |
| SNE_B24960_pgp1 | replicative DNA helicase | EFCAOE_00120, EFCAOE_03395 | **1 (Plasmid)**, 3 |
| SNE_B24950_pgp2 | virulence plasmid protein | EFCAOE_00130 | **1 (Plasmid)** |
| SNE_B24970_pgp5/parA | chromosome partitioning | EFCAOE_03160, EFCAOE_03815 | 3 |
| SNE_B24980_pgp6 | induction of IFN gamma mediated host cell response | EFCAOE_03830 | 3 |

**Table S7:** Average amino acid identity (AAI) of the Cla049 MAG with other *Simkaniaceae* and *ParaSimkaniaceae* genomes. Additional data on the reference genomes is available in Table S3.

(attached)

**Table S8:** Detailed Prokka and eggNOG-mapper annotations for *Algichlamydia australiensis* Cla049.

(attached)

**Table S9:** List of predicted secondary metabolites in *Algichlamydia australiensis* Cla049.

| **Contig name** | **Type** | **Closest biosynthetic gene cluster ID** | **Closest biosynthetic gene cluster compound** | **Core biosynthetic gene** | **Product** |
| --- | --- | --- | --- | --- | --- |
| contig_2 | NRPS-like | BGC0002711 | nostovalerolactone | EFCAOE_01065 | PlsC domain-containing protein |
| contig_3 | NRPS-like | BGC0002711 | nostovalerolactone | EFCAOE_04950 | hypothetical protein |

**Table S10:** List of hallmark chlamydial genes found in *Algichlamydia australiensis* Cla049, related to virulence processes, and their putative functions in host-chlamydiae interactions.

(attached)

**Table S11:** List of OGs present *Algichlamydia australiensis* Cla049 and absent from all other chlamydiae. The closest proteins based on AlphaFold structural predictions are provided. pLDDT: predicted local distance difference test score (high confidence if pLDDT > 70). Structures with low confidence are italicized. Only RCSB hits with pLDDT > 70 were considered.

(attached)

**Table S12:** List of genes present Algichlamydia australiensis Cla049 and absent from all other chlamydiae, and not classified into any OGs. The closest proteins based on AlphaFold structural predictions are provided. pLDDT: predicted local distance difference test score (high confidence if pLDDT > 70). Structures with low confidence are italicized. Only RCSB hits with pLDDT > 70 were considered.

(attached)

**References**

1. Butler CC, Turnham KE, Lewis AM, Nitschke MR, Warner ME, Kemp DW *et al.* Formal recognition of host-generalist species of dinoflagellate (Cladocopium, Symbiodiniaceae) mutualistic with Indo-Pacific reef corals. *J Phycol* 2023;**59**:698–711.

2. Turnham KE, Wham DC, Sampayo E, LaJeunesse TC. Mutualistic microalgae co-diversify with reef corals that acquire symbionts during egg development. *ISME J* 2021;**15**:3271–85.

3. Maire J, Girvan SK, Barkla SE, Perez-Gonzalez A, Suggett DJ, Blackall LL *et al.* Intracellular bacteria are common and taxonomically diverse in cultured and in hospite algal endosymbionts of coral reefs. *ISME J* 2021;**15**:2028–42.

4. Poppert S, Essig A, Marre R, Wagner M, Horn M. Detection and differentiation of chlamydiae by fluorescence in situ hybridization. *Appl Environ Microbiol* 2002;**68**:4081–9.

5. Wallner G, Amann R, Beisker W. Optimizing fluorescent in situ hybridization with rRNA-targeted oligonucleotide probes for flow cytometric identification of microorganisms. *Cytometry* 1993;**14**:136–43.

6. Reipert S, Gruber D, Cyran N, Schmidt B, de la Torre Noetzel R, Sancho LG *et al.* Freeze Substitution Accelerated via Agitation: New Prospects for Ultrastructural Studies of Lichen Symbionts and Their Extracellular Matrix. *Plants* 2023;**12**:4039.

7. Turnham KE, Wham DC, Sampayo E, LaJeunesse TC. Mutualistic microalgae co-diversify with reef corals that acquire symbionts during egg development. *ISME J* 2021:1–15.

8. Minh BQ, Schmidt HA, Chernomor O, Schrempf D, Woodhams MD, Von Haeseler A *et al.* IQ-TREE 2: New Models and Efficient Methods for Phylogenetic Inference in the Genomic Era. *Mol Biol Evol* 2020;**37**:1530–4.

9. Kalyaanamoorthy S, Minh BQ, Wong TKF, von Haeseler A, Jermiin LS. ModelFinder: fast model selection for accurate phylogenetic estimates. *Nat Methods* 2017;**14**:587–9.

10. Wang H-C, Minh BQ, Susko E, Roger AJ. Modeling Site Heterogeneity with Posterior Mean Site Frequency Profiles Accelerates Accurate Phylogenomic Estimation. *Syst Biol* 2018;**67**:216–35.

11. Major P, Embley TM, Williams TA. Phylogenetic Diversity of NTT Nucleotide Transport Proteins in Free-Living and Parasitic Bacteria and Eukaryotes. *Genome Biol Evol* 2017;**9**:480–7.
